# Antihemolytic and Thrombolytic Potential of *Ocimum basilicum* Seed Extract, Bioactive Compounds, and Docking With VanA Ligase in Vancomycin‐Resistant Staphylococci

**DOI:** 10.1155/jotm/6640607

**Published:** 2026-01-07

**Authors:** Khalaf F. Alsharif, Hazir Rahman

**Affiliations:** ^1^ Department of Clinical Laboratories Sciences, College of Applied Medical Sciences, Taif University, P.O. Box 11099, Taif, 21944, Saudi Arabia, tu.edu.sa; ^2^ Department of Microbiology, Abdul Wali Khan University Mardan, P.O. Box 23000, Mardan, Khyber Pakhtunkhwa, Pakistan, awkum.edu.pk

**Keywords:** ADMET, docking, in vitro and in silico assays, *O. basilicum*seeds, phytochemicals, VanA ligase, VRSA, VRSE

## Abstract

*Ocimum basilicum* is an important alternative source to explore diverse anti‐infective compounds. In the present study, aqueous seed extract of *O. basilicum* was used to identify bioactive compounds with antihemolytic, thrombolytic, antivancomycin‐resistant *Staphylococcus aureus* and antivancomycin‐resistant *S. epidermidis* activity. Anti‐VRSA and anti‐VRSE activity of *O. basilicum* seed aqueous extract was evaluated by the well diffusion assay. Hemolytic and thrombolytic activities were performed using a 96‐well plate. Phytochemical identification was done by GC‐MS. ADMET and docking analyses with VanA ligase of VRSA and VRSE were also performed. The aqueous extract showed antibacterial activity against VRSA (12 ± 0.35 mm) and VRSE (13 ± 0.11 mm) isolates. The *O. basilicum* showed significantly less hemolysis (3.7 ± 0.24%, *p* < 0.00001) of red blood cells, reflecting low cytotoxicity as compared to the control (98 ± 0.44%). The *O. basilicum* seed extract exhibited significant thrombolytic activity (4.33 ± 0.2%, *p* < 0.000429) as compared to the negative control (2 ± 0.34%). Among 23 identified compounds on GC‐MS, eight were reported for the first time in *O. basilicum* aqueous seed extract and processed for molecular docking. After favorable water solubility, pharmacokinetics, medicinal chemistry, and drug likeness, only two compounds, d‐glucopyranoside, 2,3,4,6‐di‐O‐(ethylboranediyl)‐1‐O‐methyl and 4(3,4‐dihydroxy‐2‐oxo‐butylamino) benzonitrile, were processed for molecular docking. The first one formed three hydrogen bonds with Leu‐259, Ser‐127, and His‐49 residues of the VanA ligase. The second one formed two hydrogen bonds with Ser‐161 and Val‐160 residues of the VanA ligase. d‐Glucopyranoside, 2,3,4,6‐di‐O‐(ethylboranediyl)‐1‐O‐methyl and 4(3,4‐dihydroxy‐2‐oxo‐butylamino) benzonitrile. The *O. basilicum* seed extract has potential bioactivity, and the identified compounds are novel putative VanA ligase inhibitors. Further characterization of the bioactive compounds would help to explore therapeutic targets against VRSA and VRSE.

## 1. Introduction

Plants are an essential source of medicinal molecules for human health and survival. It is reported that about one‐fourth of the drugs prescribed around the globe are plant‐based, and many people in Africa, South America, and Asia use plant‐derived medicine for their primary healthcare due to accessibility and cost‐effectiveness [1, 2].

The medicinal value of plants is mostly due to the presence of secondary bioactive compounds, including alkaloids, flavonoids, tannins, and phenolics. These bioactive compounds often have antimicrobial potential to treat infectious diseases [3, 4]. For decades, the plants’ role has been evident in traditional medicine. Among these, *Ocimum basilicum,* a sweet basil that belongs to the family Lamiaceae, is widely cultivated in the tropical regions of Africa and Asia [5, 6]. In traditional medicine, *O. basilicum* is used for the treatment of cough, bronchitis, asthma, cardiac diseases, gastrointestinal problems, and metabolic and neurocognitive disorders. Seeds of *O. basilicum* were used for treating fever, diarrhea, cold, cough, and urinary tract infections. Multiple biological activities not limited to cardioprotective effects, antimicrobial, antioxidant, anticancer, antidiabetic, and antistress have been reported for basil seed oil [5, 7, 8].

Plant‐based thrombolytic and antihemolytic potential are vital for developing effective and safe strategies to cure blood coagulations, hemolytic, and inflammatory disorders [9]. Few studies reported the antihemolytic activity of ethanolic and methanolic extracts of *O. basilicum* seeds [10, 11]. Similarly, antiplatelet aggregation and vasorelaxant effects in the presence of the *O. basilicum* aqueous extract have been reported [12, 13]; however, no data were found on the antihemolytic and thrombolytic activity of the aqueous extract of *O. basilicum* seeds.

The emergence of resistance among infectious pathogens against known antibiotics has driven the efforts to explore alternative sources of anti‐infective molecules. Furthermore, the safety, cost, and efficacy issues related to antibiotics keep plants an important alternative remedy [14]. Staphylococci are Gram‐positive bacteria and can cause minor skin infections to life‐threatening illnesses worldwide. *Staphylococcus aureus* alone was associated with above 1,000,000 deaths in 2019 [15]. Similarly, *S. epidermidis* is the causative agent of nosocomial infections [16]. Antibiotic resistance among *S. aureus* and *S. epidermidis* is a global problem. Vancomycin (VA) is a drug of choice against methicillin‐resistant *S. aureus* (MRSA) and methicillin‐resistant *S. epidermidis* (MRSE) [17, 18]. Recently, VA‐resistant *S. aureus* (VRSA) and VA‐resistant *S. epidermidis* (VRSE) have been reported [19, 20]. There are several mechanisms to acquire VA‐resistant genes among the bacteria [21]. Several *van* genes have been reported; however, the VA/Teicoplanin A‐type (VanA) ligase resistance gene is the most commonly observed gene in VRSA [22, 23]. Expression of the VanA ligase cluster of genes in *S. aureus and S. epidermidis* leads to the synthesis of peptidoglycan dipeptide precursors, D‐alanyl‐D‐lactate rather than the usual D‐Ala‐D‐Ala. VA has decreased binding affinity for D‐alanyl‐D‐lactate [23–25]. From literature mining, very few plants were screened against VA‐resistant bacteria [26]; however, no study reported *O. basilicum* seed extract activity against VRSA and VRSE, the two major nosocomial resistant pathogens.

Nowadays, gas chromatography and mass spectrometry coupled with computational methods have been successfully used to explore novel bioactive compounds from plants [27, 28]. Moreover, an *in silico* approach to predict the pharmacokinetics, medicinal chemistry, water solubility, lipophilicity, drug likeness, and toxicity, and molecular docking analysis are useful tools to quest for lead molecules [29, 30].

In the undertaken study, the aqueous seed extract of *O. basilicum* was used to identify bioactive compounds with antihemolytic, thrombolytic, anti‐VRSA, and anti‐VRSE activity. Selected compounds after ADMET analysis were screened for VanA ligase inhibitors. Further characterization of the identified compounds will help to discover therapeutic molecules against VRSA and VRSE infections.

## 2. Materials and Methods

### 2.1. Sampling

Blood culture samples of patients aged 0 days to 5 years who had been admitted to the Department of Paediatric, Mardan Medical Complex, Mardan, Pakistan, were collected. Informed consent was taken from the patient’s legal guardians. The institutional ethical committee of Abdul Wali Khan University Mardan, Pakistan, has approved the study (AWKUM/MICRO/IEC/2025/OL10).

### 2.2. *O. basilicum* Plant Collection

*O. basilicum* seeds were collected from the Mardan region of Khyber Pakhtunkhwa, Pakistan, with global positioning system coordinates of 34° 12′ 22.0428″ N and 72° 1′ 47.2800″ E. *O. basilicum* seeds were identified at the Department of Botany, Abdul Wali Khan University, Mardan, Khyber Pakhtunkhwa, Pakistan. The *O. basilicum* seeds were deposited with a voucher specimen (25‐01/OB).

### 2.3. Preparation of *O. basilicum* Seed Extracts

*O. basilicum* seed extracts were processed as previously reported [31] with few modifications. Briefly, seeds were heated at 60°C for 10 min. 100 g of powdered *O. basilicum* seeds were soaked in water (500 mL) and incubated on shaking under sterile conditions at room temperature for 1 month time. Whatman Paper No. 1 was used to filter the extract. The solvent was removed by rotary evaporation at 40°C and then stored at −80°C for downstream applications.

### 2.4. Isolation and Identification of *S. aureus* and *S. epidermidis*

Blood samples were processed for the isolation and identification of bacteria. Mannitol salt agar (MSA), Gram staining, catalase, coagulase, DNase agar, and PCR were used to identify *S. aureus*. *S. epidermidis* was identified using catalase, coagulase, and PCR assay [32, 33]. For the PCR identification of *S. epidermidis*, briefly, Chelax‐100 (BioRad, California, USA) was used for genomic DNA extraction. The resin solution was added to the bacterial pellet. The resin mixture was incubated at 95°C (10 min) and followed by centrifugation at 14,000 rpm (5 min). The supernatant was used for PCR. The *S. epidermidis* specific *rdr* gene (130 bp) primers (forward: 5‐AAG​AGC​GTG​GAG​AAA​AGT​ATC​AAG‐3; reverse: 5‐TCG​ATA​CCA​TCA​AAA​AGT​TGG‐3) were used [33]. A PCR reaction of 25 μL was preprepared with master mix (12 μL), each primer (1 μL), DNA (1 μL), and DNase‐free water (10 μL). The thermal conditions were adjusted to 95°C (10 min), followed by cycles (*n* = 35) of each 95°C (15 s), annealing at 60°C (15 s), and extension at 72°C (30 s). The final extension was performed at 72°C (15 min). The product was resolved on gel (1% agarose) and visualized on a proBLUEVIEW transilluminator (Cleaver Scientific, Taipei, Taiwan).

### 2.5. Antimicrobial Susceptibility of *S. aureus* and *S. epidermidis* Isolates From Blood

The Kirby–Bauer assay was used for the antibiogram of *S. aureus* and *S. epidermidis* isolates [34]. A bacterial inoculum of 0.5 McFarland turbidity standard was inoculated onto Mueller–Hinton agar (MHA) (Oxoid, Hampshire, UK) plates. Antibiotic‐impregnated discs (Oxoid, Hampshire, UK) of cefoxitin (FOX), cefepime (FEP), VA, and amikacin (AK) were used. The disc diffusion results of VA were further validated by the determination of the minimum inhibitory concentration of VA using a broth dilution assay for both *S. aureus and S. epidermidis* as described [35]. Briefly, VA in powdered form (500 mg) was added in 10 mL of sterile distilled water to make a 50 mg/mL concentration. Then, a 1:10 dilution in twice (0.5 mg/mL) was prepared. 640 μL from diluted antibiotic was taken, and 10 mL of sterile distilled water was added to achieve a final concentration of 32 μg/mL VA. The zone of inhibition and MICs were noted and interpreted according to guidelines [36].

### 2.6. Anti‐VRSA and Anti‐VRSE Activity of *O. basilicum* Seed Aqueous Extract

Antimicrobial activity of *O. basilicum* against VRSA and VRSE was evaluated [37]. Briefly, bacterial inoculum of 0.5 McFarland was inoculated on MHA plates. The *O. basilicum* seed aqueous extract (70 mg/mL) was added. Antibiotic discs were used as an antibiotic control, and water was used as a negative control. The plates of inoculated test micro‐organisms were incubated at 37°C for 24 h.

### 2.7. 96‐Well Plate Assay for Antihemolytic Activity of *O. basilicum* Seed Extract

The antihemolytic effect of the crude extract of *O. basilicum* was evaluated as described earlier [38]. A 5‐mL blood sample was collected and added to an EDTA tube and then centrifuged on 1500 rpm (10 min). The supernatant was discarded, and the pellet was processed. The red blood cells were rinsed with phosphate‐buffered saline (PBS) at a pH of 7.4. The cells were resuspended in 13 mL PBS. After that, the *O. basilicum* extract (70 mg/mL) was added and incubated at 37°C for 1 h. The PBS (negative control) and Triton X‐100 (positive control) were incorporated. The absorbance was measured at 540 nm to calculate the cell lysis rate. Hemolysis percentage was calculated using the formula [38].

### 2.8. Thrombolytic Activity of *O. basilicum* Seed Extract

A 5‐mL fresh human blood sample was collected to check the thrombolytic activity of the *O. basilicum* extract as described previously [39]. Briefly, the blood was added in sterile, preweighted centrifuge tubes and incubated at 37°C for 45 min. The clot was centrifuged at 2000 rpm (10 min). The clot weight was measured by deducting the weight of the empty tube from the tube containing the clot. 100 μL of *O. basilicum* extract (70 mg/mL) and 100 μL of distilled water (negative control) were added to the tubes. All the tubes were incubated at 37°C for 90 min. The clot percentage was calculated by using the formula [39]. Experiments were done in triplicate.

### 2.9. Gas Chromatography‐Mass Spectrometry Analysis for Compound Identification

Aqueous solidified extracts of *O. basilicum* were dissolved in 60% methanol as described [31]. GC‐MS (GCMS‐5977B, Agilent Technologies, California, USA) was used to identify bioactive compounds as described earlier [40]. Samples were injected via ALS front injector having a syringe size of 10 μL with an injection volume of 1 μL into a column type DB‐1 (25 m × 0.250 mm × 0.25 μm). An ESI source with 70 eV energy was used. Preinjection and postinjection washes with an injection dispense speed of 6000 μL/min were performed. Helium gas was used as the mobile phase at the rate of 1 mL/min with 8.8085 psi. The oven’s temperature was adjusted at 70°C, a final temperature of 270°C at the rate of 10°C/min with a hold time of 13 min. The MassHunter software (Agilent Technologies, California, USA) was the program utilized, and NIST11.L (National Institute of Standards Technology) served as the library. The three best hits were selected.

### 2.10. Prediction of Lead Hits

The PubChem was searched for structure parameters. SWISS‐ADME and ProTox 3 were used for the prediction of medicinal chemistry, drug likeness, pharmacokinetics, lipophilicity, and toxicity of the identified compounds [29, 41].

### 2.11. Preparation of VanA Ligase for Docking Assessment

The UniProt database (Accession ID: A0A222UBB2_STAAU) was accessed to retrieve the VanA ligase protein sequence. Protein Data Bank (PDB ID: AF‐A0A222UBB2‐F1‐model_v4) was searched for the three‐dimensional structure of VanA ligase. Briefly, the protein structure was prepared by using the QuickPrep option with default settings. The Molecular Operating Environment (MOE software, Version 2016.08) was used for molecular docking to evaluate the interactions of VanA ligase amino acid residues with the identified compounds.

### 2.12. Statistical Analysis

The results were recorded as the standard error of the mean. The Student’s *t* test was used to calculate the statistical significance. The *p* ≤ 0.05 is considered significant.

## 3. Results

### 3.1. *O. basilicum* Plant and Seed Identification

**Figure 1:**
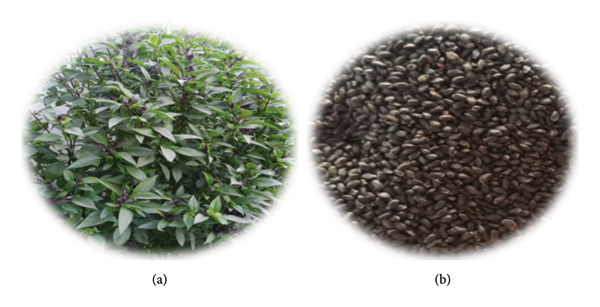
*O. basilicum* plant. (a) Whole plant and (b) seeds.

*O. basilicum* plant and seeds were identified (Figure 1).

### 3.2. Confirmation of *S. aureus* and *S. epidermidis*

**Figure 2:**
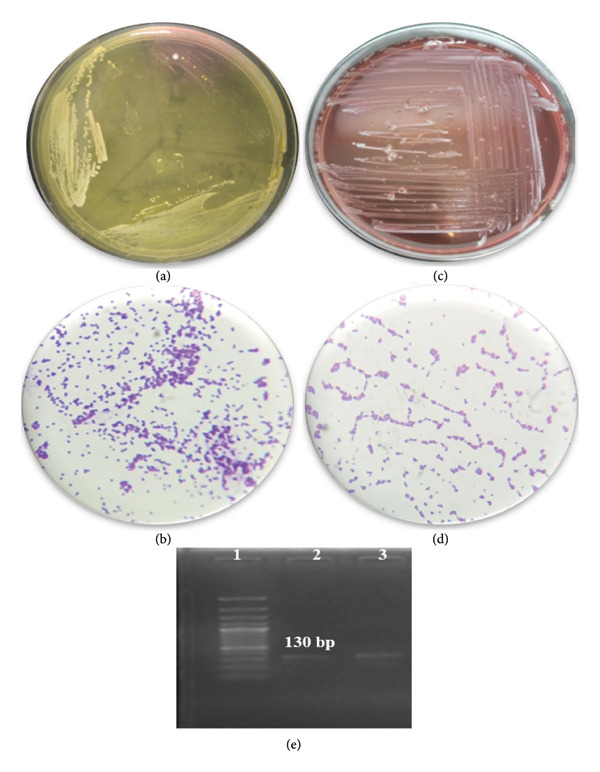
Isolation and identification of *S. aureus* and *S. epidermidis*. (a) Growth of *S. aureus* on mannitol salt agar. (b) Gram reaction of *S. aureus*. (c) Growth of *S. epidermidis* on blood agar. (d) Gram reaction of *S. epidermidis*. (e) Amplification of the *rdr* gene (130 bp) of *S. epidermidis.* 1: represent marker and 2, 3 = *rdr* positive.

The isolates were identified by biochemical and molecular methods. *S. aureus* was isolated on MSA, a selective and differential medium for *S. aureus*. On MSA, the *S. aureus* produced small yellow color colonies. Further Gram reaction, catalase, and coagulase assays were used for validation (Figures 2(a) and 2(b)). *S. epidermidis* was confirmed using a biochemical and *rdr* gene (130 bp) amplification assay (Figures 2(c), 2(d), 2(e)).

### 3.3. Antibiotic Susceptibility of VRSA and VRSE

**Figure 3:**
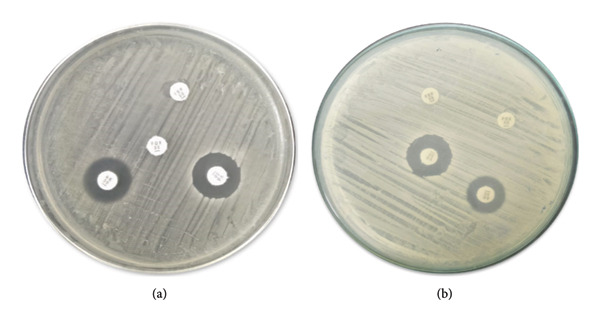
Antibiotic susceptibility of staphylococci. (a) VRSA and (b) VRSE.

*S. aureus* and *S. epidermidis* isolates exhibited resistance to FEP, FOX, AK, and VA. The disc diffusion assay was used for the VA resistance of *S. aureus* (11 ± 0.3 mm) and *S. epidermidis* (9 ± 0.5 mm). The disc diffusion findings were further validated by MIC on the broth dilution assay. The MIC of *S. aureus* was ≥ 16 μg/mL, while the MIC of *S. epidermidis* was ≥ 32 μg/mL (Figure 3, Table 1).

**Table 1:** Antibiotic susceptibility of VRSA and VRSE.

Isolate ID	Identification	Antibiotic susceptibility					Diagnosis
		Disc diffusion assay (mm)				Broth dilution assay (MIC, μg/mL)	
		Vancomycin	Cefepime	Cefoxitin	Amikacin	Vancomycin	
SA‐100	*S. aureus*	11 ± 0.3	0	0	11 ± 0.8	≥ 16	VRSA
SE‐101	*S. epidermidis*	9 ± 0.5	0	0	10 ± 0.2	≥ 32	VRSE

*Note:* ±: standard error of the given value. As per CLSI guidelines for disc diffusion assay, amikacin ≤ 14 mm is considered resistant for *S. aureus* and *S. epidermidis.* Vancomycin ≤ 16 mm and ≤ 10 mm are considered resistant for *S. aureus* and *S. epidermidis,* respectively. As per CLSI guidelines for broth dilution assay, the MIC ≥ 16 μg/mL and ≥ 32 μg/mL are considered resistant for *S. aureus and S. epidermidis,* respectively.

### 3.4. Antibacterial, Hemolytic, and Thrombolytic Activity of *O. basilicum* Seed Extract

**Figure 4:**
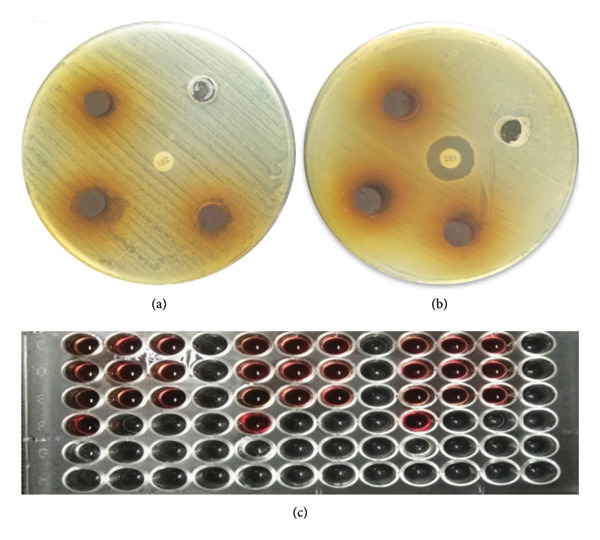
Anti‐VRSA, anti‐VRSE, and hemolytic activity of the aqueous extract of *O. basilicum.* (a) Anti‐VRSA activity; upper right: negative control, middle: antibiotic control (vancomycin). (b) Anti‐VRSE activity; upper right: negative control, and middle: antibiotic control (vancomycin). (c) Hemolytic activity of the aqueous extract of *O. basilicum:* (C, D) blood with plant extract in triplicate, (E) positive control (triplicate, blood + Triton X‐100), (F) Negative control triplicate (PBS + blood).

*O. basilicum* seed extract (70 mg/mL) exhibited potential activity against VRSA (12 ± 0.35 mm) and VRSE (13 ± 0.11 mm) (Figures 4(a) and 4(b)). The hemolytic activity was significantly (*p* < 0.00001) decreased in the *O. basilicum* extract (3.7 ± 0.24%) as compared to the positive control (98 ± 0.44%) (Figure 4(c)). The thrombolytic activity was significantly (*p* < 0.000429) increased in the *O. basilicum* extract (4.33 ± 0.2%) as compared to the negative control (2 ± 0.34%). The antibacterial, hemolytic, and thrombolytic activities are listed in Table 2.

**Table 2:** Anti‐VRSA, anti‐VRSE, hemolytic, and thrombolytic activity of the aqueous extract of *O. basilicum* seeds.

Antibacterial activity (mm)		Hemolytic activity (%)				Thrombolytic activity (%)		
*O. basilicum* extract (70 mg/mL)		*O. basilicum* (70 mg/mL)	Positive control	^∗^Negative control	*P* value	*O. basilicum* (70 mg/mL)	Negative control	*p* value
VRSA (mm)	VRSE (mm)							
12 ± 0.35	13 ± 0.11	3.7 ± 0.24	98 ± 0.44	0	< 0.00001	4.33 ± 0.2	2 ± 0.34	< 0.000429

*Note:* Water as solvent control. Positive control: blood + Triton X‐100.

^∗^Negative control: PBS + blood. Negative control: distilled water. *p* ≤ 0.05 is considered significant.

### 3.5. Identification of Bioactive Compounds

**Figure 5:**
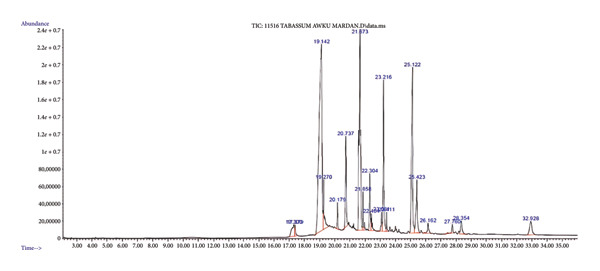
GC‐MS chromatogram of bioactive aqueous extract of *O. basilicum* seeds.

The unknown spectra were compared with the known spectra in the in the NIST library. The overall GC‐MS spectra with time and abundance are provided in Figure 5. After a total of 23 compounds were identified on GC‐MS. The detailed spectral details, compound names, RT, *m/z* ratios, and CAS numbers are provided (Table 3, Supporting Figure S1).

**Table 3:** Bioactive compounds identified on the GC‐MS analysis of the aqueous extract of *O. basilicum* seeds.

S. no.	Compound	Peak no.	Retention time (min)	CAS no.	*m/z*
1.	n‐Hexadecanoic acid	1, 2	17.300	000057‐10‐3	*60.00*
2.	6‐Octadecenoic acid	3, 4	19.142	1000336‐66‐8	*69.10*
3.	Oleic acid	3, 6	19.142	000112‐80‐1	*41.10*
4.	8‐Heptadecene	5	20.179	002579‐04‐6	*57.10*
5.	1,15‐Pentadecanedioic acid	5, 7	20.179	001460‐18‐0	*55.10*
6.	cis‐13‐Eicosenoic acid	6	20.737	017735‐94‐3	*83.10*
7.	Cyclohexanecarboxylic acid, undecyl ester	7, 14, 17	21.673	094107‐44‐5	*67.10*
8.	Palmitoyl chloride	8	21.858	000112‐67‐4	*98.10*
9.	d‐Glucopyranoside, 2,3,4,6‐di‐O‐(ethylboranediyl)‐1‐O‐methyl	8, 13	21.858	1000149‐94‐5	*57.10*
10.	2‐Dodecylcyclohexanone	8	21.858	015674‐95‐0	*55.10*
11.	Erucic acid	9	22.304	000112‐86‐7	*83.10*
12.	cis‐10‐Nonadecenoic acid	9	22.304	073033‐09‐7	*41.10*
13.	Bicyclo[5.3.0]decane (cis)	10	22.404	016189‐46‐1	*83.10*
14.	4(3,4‐Dihydroxy‐2‐oxo‐butylamino) benzonitrile	11	23.081	1000188‐22‐9	*69.10*
15.	Bis[2‐(cinnamoyloxy)‐1‐naphthyl]methane	11	23.081	293761‐81‐6	*117.00*
16.	Adipic acid, butyl cycloheptyl ester	12	23.216	1000324‐71‐6	*69.10*
17.	4‐Trifluoromethylbenzoic acid. 4‐Hexadecyl ester	15, 18, 19	25.423	1000283‐03‐4	*69.10*
18.	Decyl sulfide	15	25.423	000693‐83‐4	*83.10*
19.	1,14‐Docosanediol	16	26.162	004452‐45‐3	*69.10*
20.	11‐Hexadecen‐1‐ol, acetate, (Z)‐	16	26.162	034010‐21‐4	*83.10*
21.	Bicyclo[10.8.0]eicosane, cis‐	16	26.162	1000155‐82‐2	*26.162*
22.	Propanamide, N‐(5,7‐dimethyl‐1,8‐naphthyridin‐2‐yl)‐	20	32.928	296244‐68‐3	*160.00*
23.	Coumarine‐3‐carbohydrazide, N2‐(1‐methylethenylideno)‐	20	32.928	204185‐63‐7	*83.10*

*Note:* *m/z:* mass‐to‐charge ratio.

### 3.6. Druggable Characteristics of Bioactive Compounds

**Figure 6:**
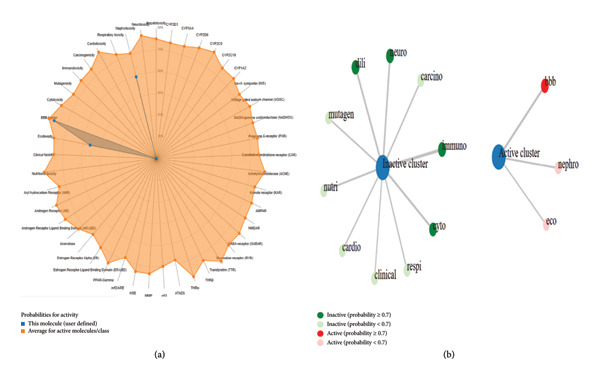
Predicted toxicity profile of d‐glucopyranoside, 2,3,4,6‐di‐O‐(ethylboranediyl)‐1‐O‐methyl. (a) The toxicity radar chart illustrated the toxicity results. (b) The network chart.

**Figure 7:**
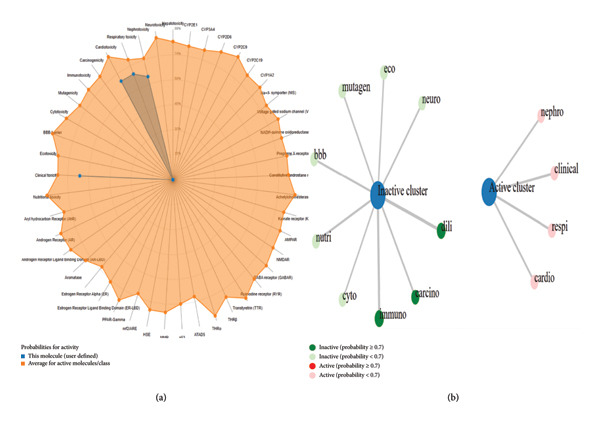
Predicted toxicity profile of 4 (3,4‐dihydroxy‐2‐oxo‐butylamino) benzonitrile. (a) The toxicity radar chart. (b) The network chart.

After extensive literature search, among the 23 compounds, eight compounds were for the first time reported in the *O. basilicum* seed extract. These eight compounds were processed for ADME, toxicity, medicinal chemistry, and drug‐likeness characteristics (Table 4, and Figures 6 and 7).

**Table 4:** Druggable compounds of the *O. basilicum* extract.

Description	Characteristics of druggable compounds							
	cis‐13‐Eicosenoic acid	d‐Glucopyranoside, 2,3,4,6‐di‐O‐(ethylboranediyl)‐1‐O‐methyl	2‐Dodecylcyclohexanone	Erucic acid	cis‐10‐Nonadecenoic acid	4(3,4‐Dihydroxy‐2‐oxo‐butylamino) benzonitrile	Decyl sulfide	1,14‐Docosanediol
*1. Physicochemical profile*								
M.W (g/mol)	310.51	269.89	266.46	338.57	296.49	220.22	314.61	342.60
Atoms heavy (*n*)	22	19	19	24	21	16	21	24
Atoms heavy aromatic (*n*)	0	0	0	0	0	6	0	0
Fraction Csp3	0.85	1	0.94	0.86	0.84	0.27	1	1
Bonds rotatable (*n*)	17	3	11	19	16	5	18	20
Acceptors H‐bond (*n*)	2	6	1	2	2	4	0	2
Donors hydrogen	1	0	0	1	1	3	0	2
Refractivity (M)	99.55	68.78	86.73	109.17	94.74	57.41	105.85	110.19
Å^2 (^TPSA)	37.30	55.38	17.07	37.30	37.30	93.35	25.30	40.46

*2. Lipophilicity*								
MLOGP	5.03	−1.15	4.40	5.47	4.80	−0.61	6.47	4.91
iLOGP	4.59	0	4.36	5.07	4.35	1.37	5.68	5.58
WLOGP	6.89	0.57	6.06	7.67	6.50	−0.30	8	6.77
XLOGP3	8.72	1.49	7.28	9.81	8.18	0.63	9.88	8.47
Log *P*_*o*/*w*_ consensus	6.41	−0.22	5.66	7.14	6.04	0.37	7.63	6.66

*3. Water solubility*								
ESOL	−6.14	−2.25	−5.35	−6.87	−5.78	−1.55	−6.83	−5.98
Solubility	Poorly soluble	Soluble	Moderately soluble	Poorly soluble	Moderately soluble	Very soluble	Poorly soluble	Moderately soluble
Solubility (mg/mL; mol/L)	2.27 × 10^−4^; 7.30 × 10^−7^	1.50 × 10; 5.57 × 10^−4^	1.18 × 10^−3^; 4.44 × 10^−6^	4.62 × 10^−5^; 1.36 × 10^−7^	4.97 × 10^−4^; 1.68 × 10^−6^	6.21 × 10; 2.82 × 10^−2^	4.69 × 10^−5^; 1.49 × 10^−7^	3.59 × 10^−4^; 1.05 × 10^−6^
Log *S* (Ali)	−9.38	−2.26	−7.46	−10.51	−8.82	−2.17	−10.33	−9.19
Solubility class	Poorly soluble	Soluble	Poorly soluble	Insoluble	Poorly soluble	Soluble	Insoluble	Poorly soluble
Solubility (mg/mL; mol/L)	1.28 × 10^−7^; 4.14 × 10^−10^	1.48 × 10 5.4910^−3^	9.15 × 10^−6^; 3.43 × 10^−8^	1.04 × 10^−8^; 3.06 × 10^−11^	4.46 × 10^−7^; 1.50 × 10^−9^	1.51 × 10; 6.83 × 10^−3^	1.45 × 10^−8^; 4.62 × 10^−11^	2.21 × 10^−7^; 6.45 × 10^−10^
SILICOS‐IT	−6.19	−0.88	−6.05	−6.99	−5.79	−2.20	−8.03	−7.23
Solubility class	Poorly soluble	Soluble	Poorly soluble	Poorly soluble	Moderately soluble	Soluble	Poorly soluble	Poorly soluble
Solubility (mg/mL; mol/L)	2.00 × 10^−4^; 6.44 × 10^−7^	3.58 × 10; 1.33 × 10^−1^	2.39 × 10^−4^; 8.97 × 10^−7^	3.50 × 10^−5^; 1.04 × 10^−7^	4.78 × 10^−4^; 1.61 × 10^−6^	1.39 × 10; 6.31 × 10^−3^	2.92 × 10^−6^; 9.27 × 10^−49^	2.03 × 10^−5^; 5.93 × 10^−8^

*4. Pharmacokinetics*								
Skin permeability (cm/s)	−2.00	−6.89	−2.76	−1.40	−2.30	−7.20	−1.20	−2.38
Blood–brain barrier (permeability)	No	No	No	No	No	No	No	No
Gastrointestinal absorption	Low	High	High	Low	High	High	Low	High
P‐g proteins substrate	No	No	No	No	No	No	No	No
CYP2D6	No	No	No	No	No	No	No	No
CYP1A2	Yes	No	Yes	Yes	Yes	No	Yes	Yes
CYP2C19	No	No	No	No	No	No	No	No
CYP2C9	Yes	No	Yes	No	Yes	No	No	No
CYP3A4	No	No	No	No	No	No	No	No
CYP2C19	No	No	No	No	No	No	No	No

*5. Drug likeness*								
Lipinski	Yes; 1	Yes; 0	Yes; 1 n	Yes; 1	Yes; 1	Yes; 0	Yes; 1	Yes; 1
Muegge	No; 2 s	Yes	No; 2	No; 2	No; 2	Yes	No; 3	No; 2
Ghose	No; 1	Yes	No; 1	No; 1	No; 1	Yes	No; 1	No; 1
Egan	No; 1	Yes	No; 1	No; 1	No; 1	Yes	No; 1	No; 1
Veber	No; 1	Yes	No; 1	No; 1	No; 1	Yes	No; 1	No; 1
Bioavailability (score)	0.85	0.55	0.55	0.85	0.85	0.55	0.55	0.55

*6. Medicinal chemistry*								
Lead likeness violation	No; 2	Yes	No; 2	No; 2	No; 2	No; 1	No; 2	No; 2
Brenk alert	1 alert	1 alert	0	1 alert	1 alert	0	0	0
PAINS alert	0	0	0	0	0	0	0	0
Synthetic accessibility	3.30	4.85	3.01	3.53	3.18	2.15	3.82	3.48

*7. Toxicity*								
LD50 predicted (mg/kg)	48	23000	500	48	48	1200	4700	1000
Toxicity class	2	6	4	2	2	4	5	4

### 3.7. Molecular Docking of Two Compounds With VanA Ligase

**Figure 8:**
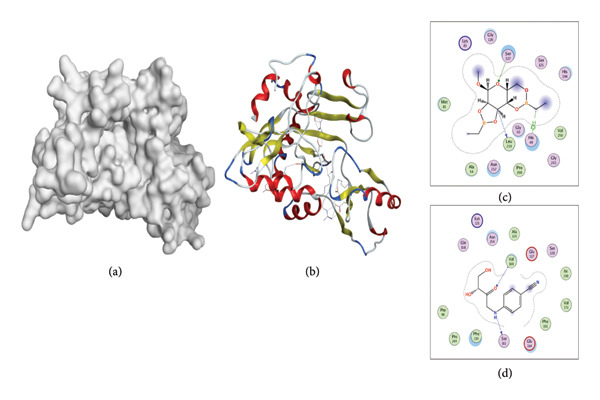
Docking analysis of VanA ligase protein with d‐glucopyranoside, 2,3,4,6‐di‐O‐(ethylboranediyl)‐1‐O‐methyl and 4(3,4‐dihydroxy‐2‐oxo‐butylamino) benzonitrile. (a) Surface map of VanA ligase protein. (b) Molecular structure of VanA ligase. (c) Enzyme VanA ligase interaction with d‐glucopyranoside, 2,3,4,6‐di‐O‐(ethylboranediyl)‐1‐O‐methyl. (d) Enzyme VanA ligase interaction with 4(3,4‐dihydroxy‐2‐oxo‐butylamino) benzonitrile.

Two compounds, d‐glucopyranoside, 2,3,4,6‐di‐O‐(ethylboranediyl)‐1‐O‐methyl and 4(3,4‐dihydroxy‐2‐oxo‐butylamino) benzonitrile, were selected for molecular docking after the prediction of feasible water solubility, pharmacokinetics, medicinal chemistry, and drug likeness. The d‐glucopyranoside, 2,3,4,6‐di‐O‐(ethylboranediyl)‐1‐O‐methyl formed three hydrogen bonds with Leu‐259, Ser‐127, and His‐49 residues of the VanA ligase. 4(3,4‐Dihydroxy‐2‐oxo‐butylamino) benzonitrile formed two hydrogen bonds with Ser‐161 and Val‐160 residues of the VanA ligase. These ligand–enzyme interactions would have a possible role in regulating enzyme activity (Table 5, Figure 8).

**Table 5:** Docking analysis of two compounds with VanA ligase protein.

S. no.	Compound	Ligand	Receptor	Association	Bond distance	Energy (kcal/mol)
1	d‐Glucopyranoside, 2,3,4,6‐di‐O‐(ethylboranediyl)‐1‐O‐methyl	C 7	O Leu‐259 (A)	H‐donor	3.27	−0.7
		O 1	OG Ser‐127 (A)	H‐acceptor	3.17	−0.8
		C 14	5‐ring His‐49 (A)	H‐pi	3.77	−1.0

2	4 (3,4‐Dihydroxy‐2‐oxo‐butylamino) benzonitrile	N 4	O Ser‐161 (A)	H‐donor	3.19	−1.0
		O 2	N Val‐160 (A)	H‐acceptor	2.97	−1.3

## 4. Discussion

Staphylococci are Gram‐positive bacteria and can cause several infections in humans worldwide. *S. aureus* and *S. epidermidis* are the common Staphylococci causing nosocomial infections. The emergence of antibiotic resistance among *S. aureus* and *S. epidermidis* is a global challenge to public health [42, 43]. In the acquisition of MRSA and MRSE, VA is a drug of last resort to treat [17, 18]. Recently, VRSA and VRSA have been reported, which pose a real threat to available antibiotics [19, 20]. VRSA and VRSE often express VanA gene, which encodes VanA ligase. The VanA ligase expression leads to the synthesis of D‐alanyl‐D‐lactate, an unusual peptidoglycan dipeptide precursor, and thus exhibits resistance to VA [23, 24]. Given the alarming antibiotic resistance and their efficacy and safety issues, medicinal plants are an important alternative source.

Among the medicinal plants, *O. basilicum* is used to treat throat infection, bronchitis, asthma, cardiac diseases, and gastrointestinal problems. Similarly, *O. basilicum* seeds were used for treating diarrhea, fever, and urinary tract infections [5, 7]. The pharmacological importance is attributed to the presence of alkaloids, flavonoids, and saponins [5].

In the current study, *O. basilicum* aqueous seed extracts were processed for antihemolytic and thrombolytic activity, anti‐VRSA and anti‐VRSE activity, bioactive compound identification, ADMET, and VanA ligase docking analysis.

Plant‐based antihemolytic and anticoagulation activity are important for developing effective strategies to cure blood‐related disorders [9]. *O. basilicum* aqueous seed extract exhibited significant antihemolytic activity (3.7 ± 0.24%, *p* < 0.00001), which reflects low cytotoxicity toward red blood cells and might be of safer nature in medicinal ingredients. Another study on oil from the *O. basilicum* flower reported a hemolytic effect to 3.90% [44]. Similarly, significant (*p* < 0.000429) thrombolytic activity was noted. Previous work on the whole plant *O. basilicum* aqueous extract showed a vasorelaxant and antiplatelet aggregation effect [13]. Further detailed investigations are required to conclude its therapeutic role in blood and cardiovascular diseases.

*O. basilicum* seed extract exhibited anti‐VRSA and anti‐VRSE activity. A previous study reported *O. basilicum* leaves as antimicrobial against *S. aureus*, *Pseudomonas aeruginosa*, and *Escherichia coli* [45]; however, the current study reported for the first time the anti‐VRSA and anti‐VRSE activity of *O. basilicum.* These findings would help to further explore the therapeutic potential of *O. basilicum* against resistant pathogens causing human infections.

The bioactive extract of *O. basilicum* seeds was processed for GC‐MS analysis. A total of 23 compounds were identified. A recent study reported the diverse phytochemical content in the leaves of *O. basilicum* [46]. Further use of advanced analytical techniques would help to explore more compounds having medicinal worth.

Computational approaches, including ADMET, physiochemical, and drug‐likeness characteristics, are important to quest novel antibacterial compounds [28, 29, 41]. From the literature search, among the 23 compounds, eight were reported for the first time in *O. basilicum* seeds. Eight compounds were processed for ADMET analysis, medicinal chemistry, and drug‐likeness characteristics. Based on water solubility, Lipinski’s rule of five, medicinal chemistry, and ADMET data, d‐glucopyranoside, 2,3,4,6‐di‐O‐(ethylboranediyl)‐1‐O‐methyl and 4(3,4‐dihydroxy‐2‐oxo‐butylamino) benzonitrile were processed for docking analysis. Molecular docking tools are widely used to investigate ligand–receptor interaction [30]. In this study, VanA ligase of *S. aureus* was used as a ligand molecule. VanA ligase expression in *S. aureus and S. epidermidis* triggers the synthesis of an unusual dipeptide D‐alanyl‐D‐lactate to which VA has decreased binding affinity and leads to the emergence of VRSA and VRSE [23, 24]. Docking of d‐glucopyranoside, 2,3:4,6‐di‐O‐(ethylboranediyl)‐1‐O‐methyl and 4(3,4‐dihydroxy‐2‐oxo‐butylamino) benzonitrile exhibited interactions with several amino acid residues of the VanA ligase of VRSA.

In the current study, aqueous extracts of *O. basilicum* seeds were evaluated against VRSA and VRSE, and for *in silico* analysis; however, for further validation of the identified bioactive compounds, in vitro and in vivo studies are required to conclude their role as anti‐infective agents.

## 5. Conclusion

The aqueous seed extract of *O*. *basilicum* demonstrated significant anti‐VRSA, anti‐VRSE, antihemolytic, and thrombolytic activities. Among 23 identified compounds, eight were reported for the first time in the *O. basilicum* aqueous seed extract. The d‐glucopyranoside, 2,3,4,6‐di‐O‐(ethylboranediyl)‐1‐O‐methyl and 4(3,4‐dihydroxy‐2‐oxo‐butylamino) benzonitrile showed potential VanA ligase inhibition. Further characterization of the identified compounds will help to explore anti‐VRSA and anti‐VRSE compounds to treat the infections caused by Staphylococci.

## Disclosure

All the authors have read and agreed to the published version of the manuscript.

## Conflicts of Interest

The authors declare no conflicts of interest.

## Author Contributions

Conceptualization, methodology, resources, validation, formal analysis, writing–original draft, and writing–review and editing: Khalaf F. Alsharif. Conceptualization, methodology, project administration, supervision, formal analysis, writing–original draft, and writing–review and editing: Hazir Rahman.

## Funding

This research was funded by Taif University, Taif, Saudi Arabia (TU‐DSPP‐2024‐26).

## Supporting Information

GC‐MS raw spectral data are provided as Supporting Figure S1.

## Supporting information


**Supporting Information** Additional supporting information can be found online in the Supporting Information section.

## Data Availability

The data that support the findings of this study are available from the corresponding author upon reasonable request.
