# Supplementary material for: Antihemolytic and Thrombolytic Potential of Ocimum basilicum Seed Extract, Bioactive Compounds, and Docking With VanA Ligase in Vancomycin‐Resistant Staphylococci
Source: J Trop Med. 2026 Jan 7;2026:6640607. doi: 10.1155/jotm/6640607 (PMC12775832; doi:10.1155/jotm/6640607)
Supplement: Supplementary file 1 — Supporting Information Additional supporting information can be found online in the Supporting Information section. [file JOTM-2026-6640607-s001.pdf]

Library Search Report

```
Data Path : D:\GCMS RESULTS DATA\
Data File : 11516 TABASSUM AWKU MARDAN.D
Acq On    : 25 Mar 2025  11:22
Operator  : CRL
Sample    : SAMPLE 1
Misc      :
ALS Vial  : 1   Sample Multiplier: 1

Search Libraries:   C:\Database\NIST11.L             Minimum Quality:   0

Unknown Spectrum:   Apex
Integration Events: ChemStation Integrator - events.e
```

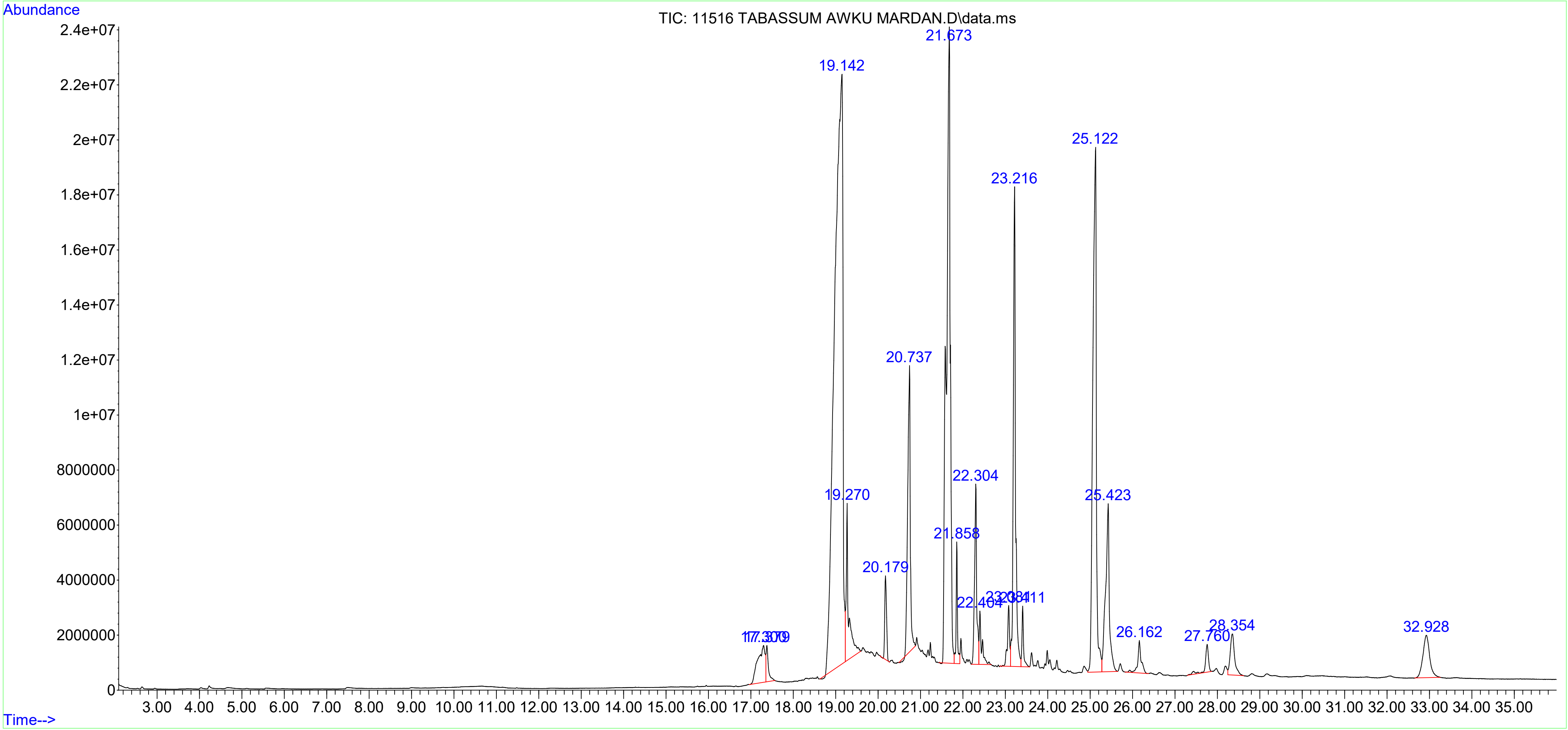

Library Search Report - ChemStation Integrator

Unknown Spectrum based on Apex

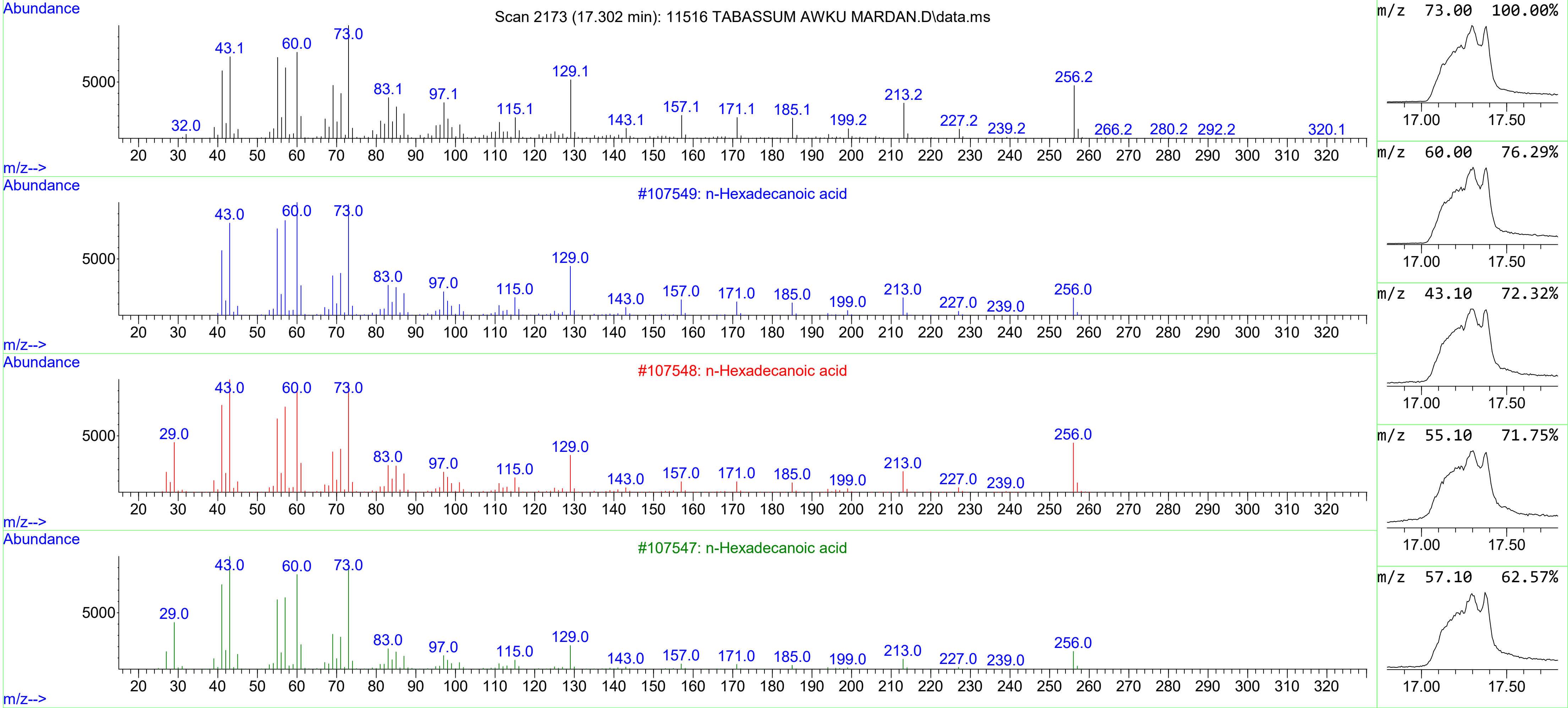

Data File: D:\GCMS RESULTS DATA\11516 TABASSUM AWKU MARDAN.D
Sample : SAMPLE 1

Peak Number: 1 at 17.300 min Area: 159672864 Area % 1.90

The 3 best hits from each library.	Ref\#	CAS\#	Qual
C:\Database\NIST11.L			
1 n-Hexadecanoic acid	107549	000057-10-3	99
2 n-Hexadecanoic acid	107548	000057-10-3	98
3 n-Hexadecanoic acid	107547	000057-10-3	96

Unknown Spectrum based on Apex

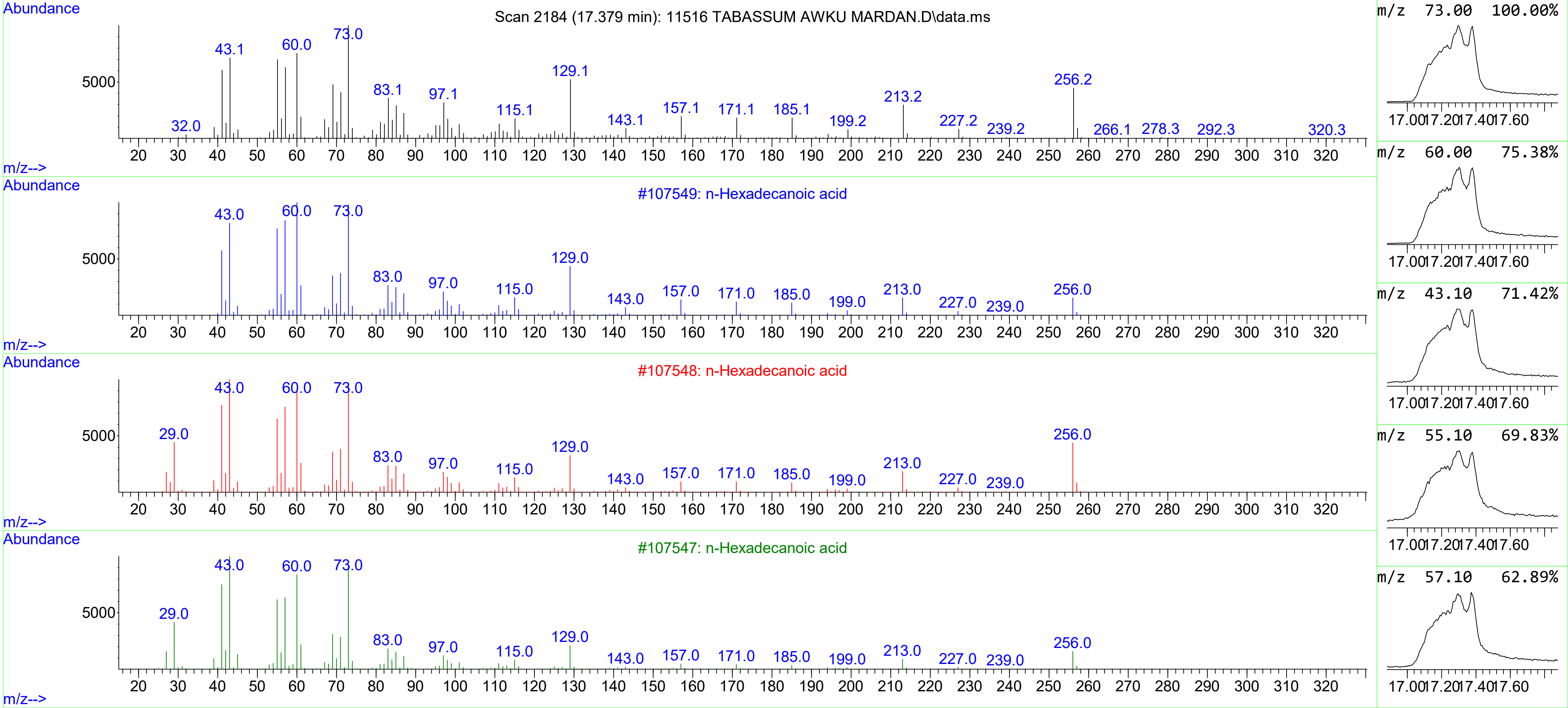

Data File: D:\GCMS RESULTS DATA\11516 TABASSUM AWKU MARDAN.D
Sample : SAMPLE 1

Peak Number: 2 at 17.379 min Area: 51574478 Area % 0.61

The 3 best hits from each library.	Ref\#	CAS\#	Qual
C:\Database\NIST11.L			
1 n-Hexadecanoic acid	107549	000057-10-3	99
2 n-Hexadecanoic acid	107548	000057-10-3	98
3 n-Hexadecanoic acid	107547	000057-10-3	97

Library Search Report - ChemStation Integrator

Unknown Spectrum based on Apex

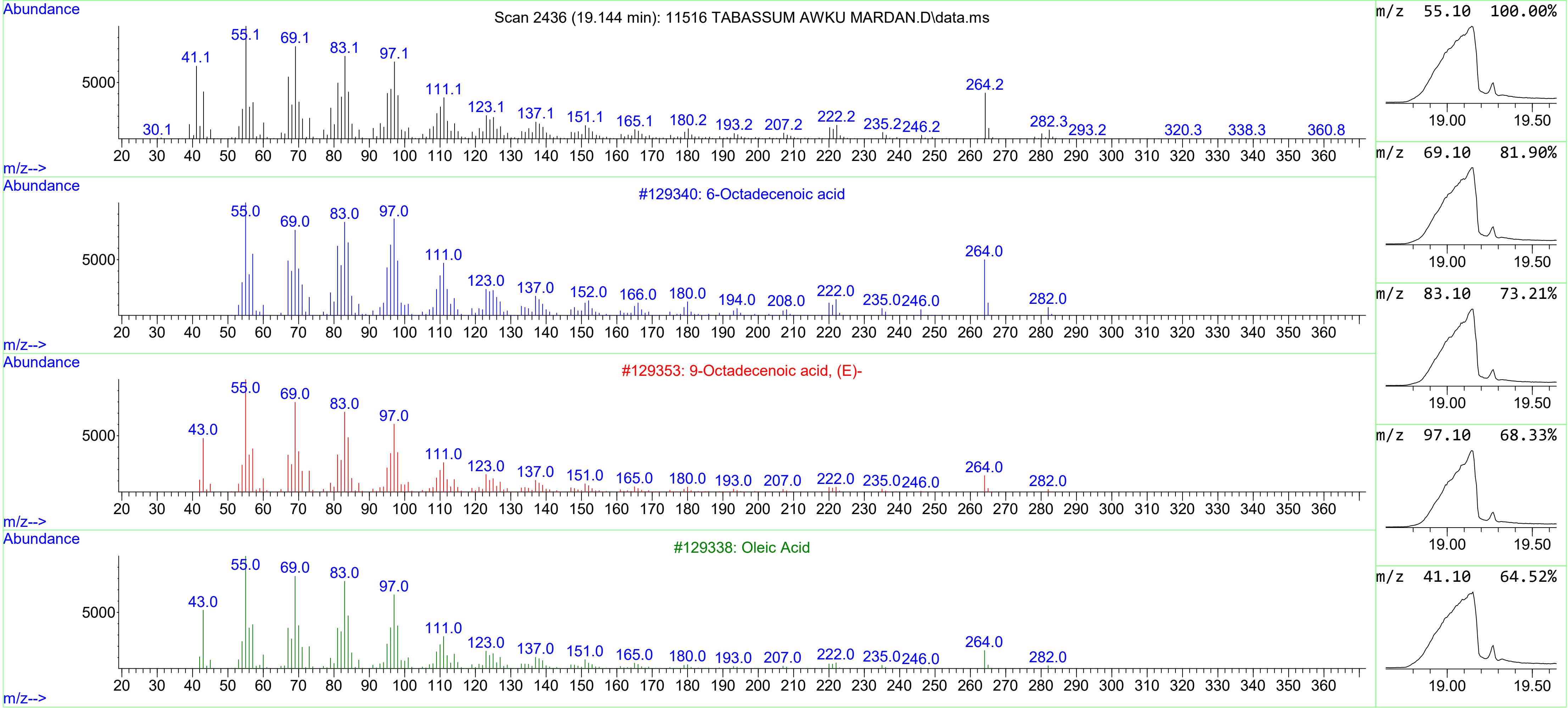

Data File: D:\GCMS RESULTS DATA\11516 TABASSUM AWKU MARDAN.D
Sample : SAMPLE 1

Peak Number: 3 at 19.142 min Area: 2924765483 Area % 34.81

The 3 best hits from each library.	Ref#	CAS#	Qual
C:\Database\NIST11.L			
1 6-Octadecenoic acid	129340	1000336-66-8	99
2 9-Octadecenoic acid, (E)-	129353	000112-79-8	99
3 Oleic Acid	129338	000112-80-1	99

Library Search Report - ChemStation Integrator

Unknown Spectrum based on Apex

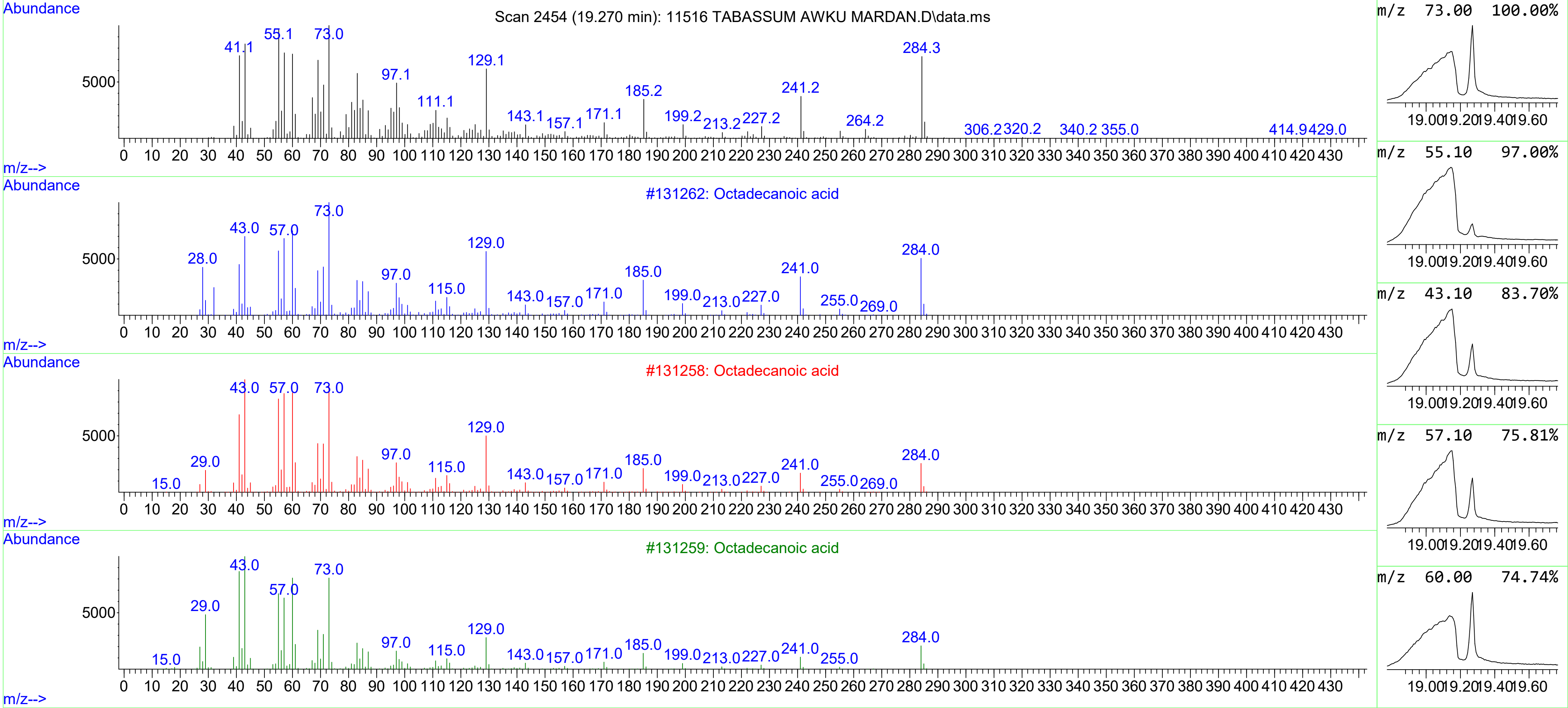

Data File: D:\GCMS RESULTS DATA\11516 TABASSUM AWKU MARDAN.D
Sample : SAMPLE 1

Peak Number: 4 at 19.270 min Area: 231628414 Area % 2.76

The 3 best hits from each library.	Ref\#	CAS\#	Qual
C:\Database\NIST11.L			
1 Octadecanoic acid	131262	000057-11-4	99
2 Octadecanoic acid	131258	000057-11-4	97
3 Octadecanoic acid	131259	000057-11-4	93

Unknown Spectrum based on Apex

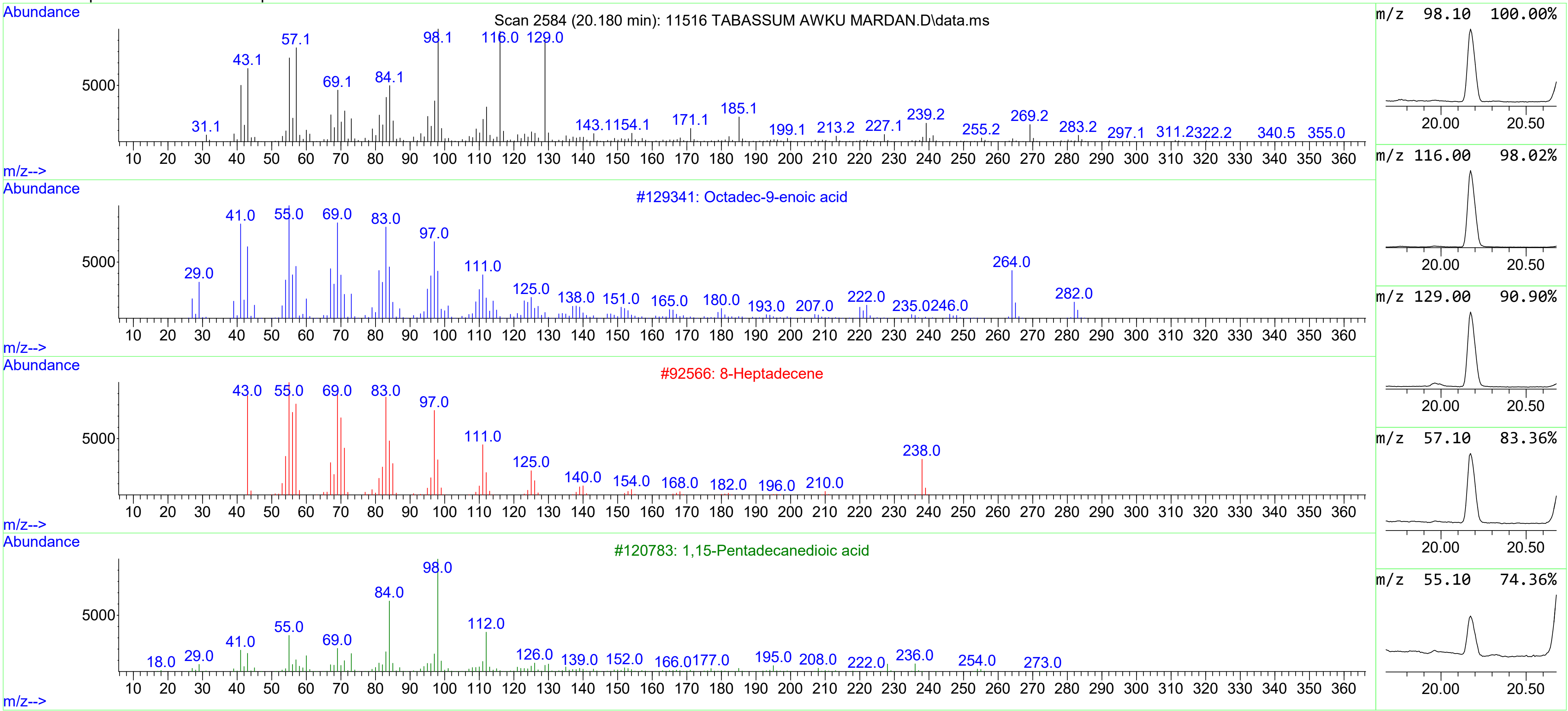

Data File: D:\GCMS RESULTS DATA\11516 TABASSUM AWKU MARDAN.D
Sample : SAMPLE 1

Peak Number: 5 at 20.179 min Area: 90622220 Area % 1.08

The 3 best hits from each library.	Ref\#	CAS\#	Qual
C:\Database\NIST11.L			
1 Octadec-9-enoic acid	129341	1000190-13-7	45
2 8-Heptadecene	92566	002579-04-6	41
3 1,15-Pentadecanedioic acid	120783	001460-18-0	38

Unknown Spectrum based on Apex

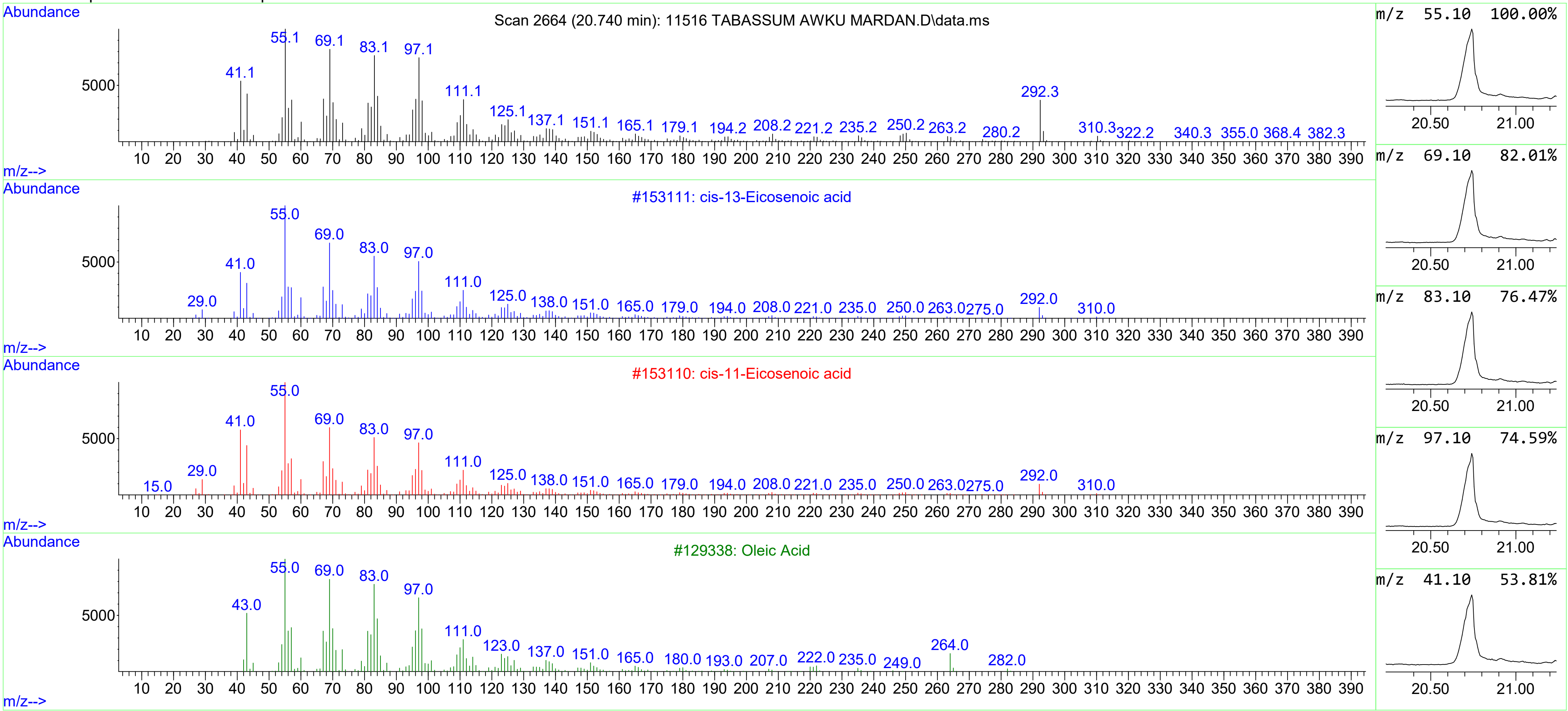

Data File: D:\GCMS RESULTS DATA\11516 TABASSUM AWKU MARDAN.D
Sample : SAMPLE 1

Peak Number: 6 at 20.737 min Area: 438112261 Area % 5.21

The 3 best hits from each library.	Ref\#	CAS\#	Qual
C:\Database\NIST11.L			
1 cis-13-Eicosenoic acid	153111	017735-94-3	99
2 cis-11-Eicosenoic acid	153110	005561-99-9	99
3 Oleic Acid	129338	000112-80-1	94

Unknown Spectrum based on Apex

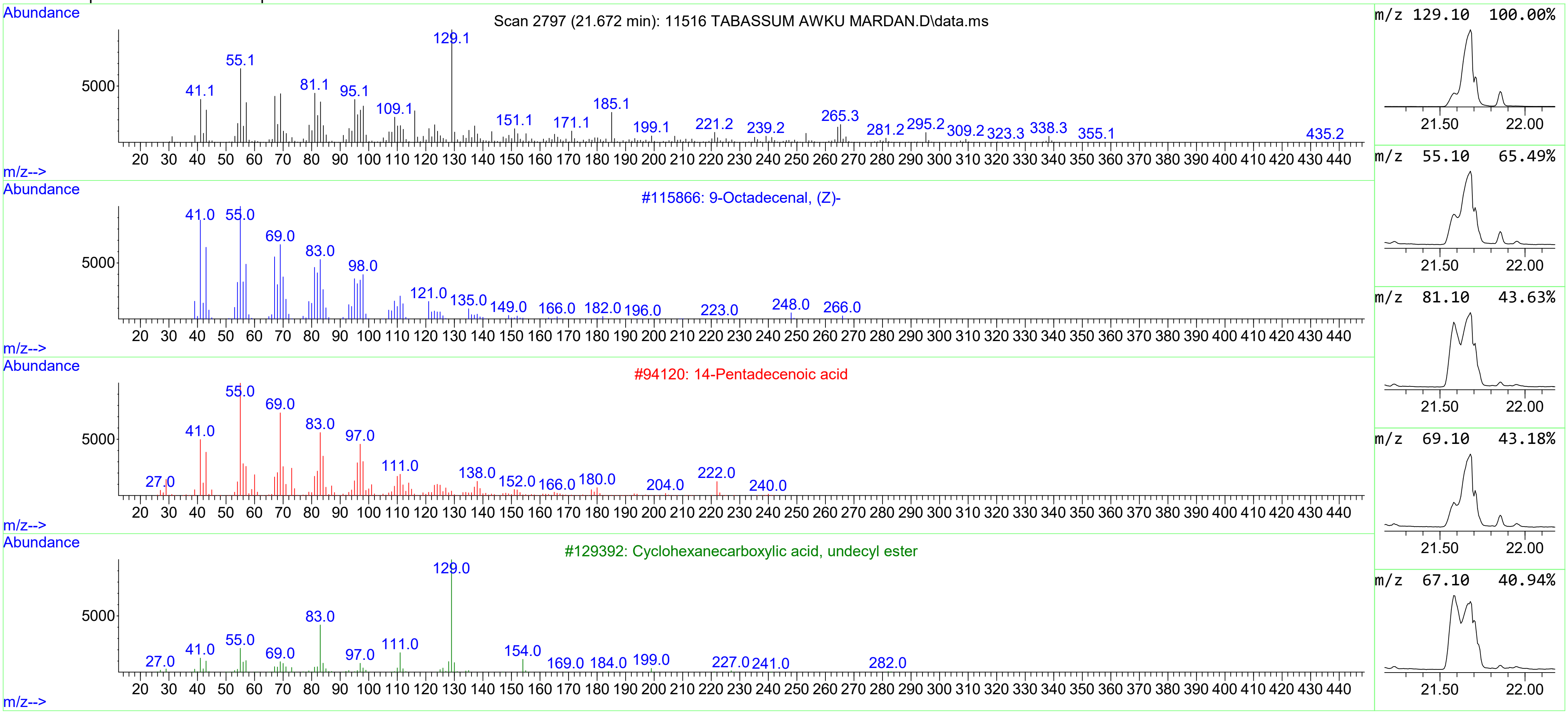

Data File: D:\GCMS RESULTS DATA\11516 TABASSUM AWKU MARDAN.D
Sample : SAMPLE 1

Peak Number: 7 at 21.673 min Area: 1469489012 Area % 17.49

The 3 best hits from each library.	Ref\#	CAS\#	Qual
C:\Database\NIST11.L			
1 9-Octadecenal, (Z)-	115866	002423-10-1	94
2 14-Pentadecenoic acid	94120	017351-34-7	43
3 Cyclohexanecarboxylic acid, unde...	129392	094107-44-5	38

Unknown Spectrum based on Apex

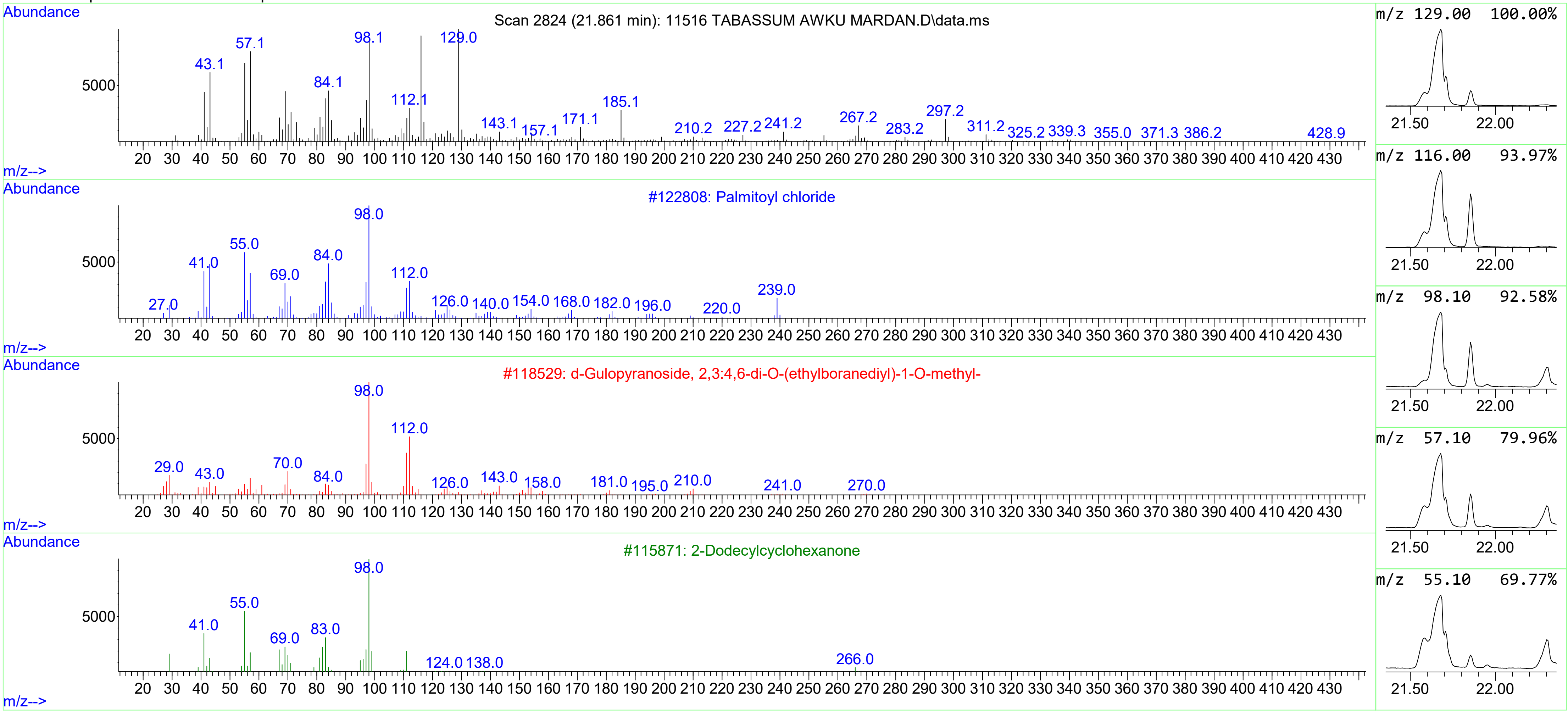

Data File: D:\GCMS RESULTS DATA\11516 TABASSUM AWKU MARDAN.D
Sample : SAMPLE 1

Peak Number: 8 at 21.858 min Area: 96450305 Area % 1.15

The 3 best hits from each library.	Ref\#	CAS\#	Qual
C:\Database\NIST11.L			
1 Palmitoyl chloride	122808	000112-67-4	41
2 d-Gulopyranoside, 2,3:4,6-di-O-(...	118529	1000149-94-5	25
3 2-Dodecylcyclohexanone	115871	015674-95-0	18

Unknown Spectrum based on Apex

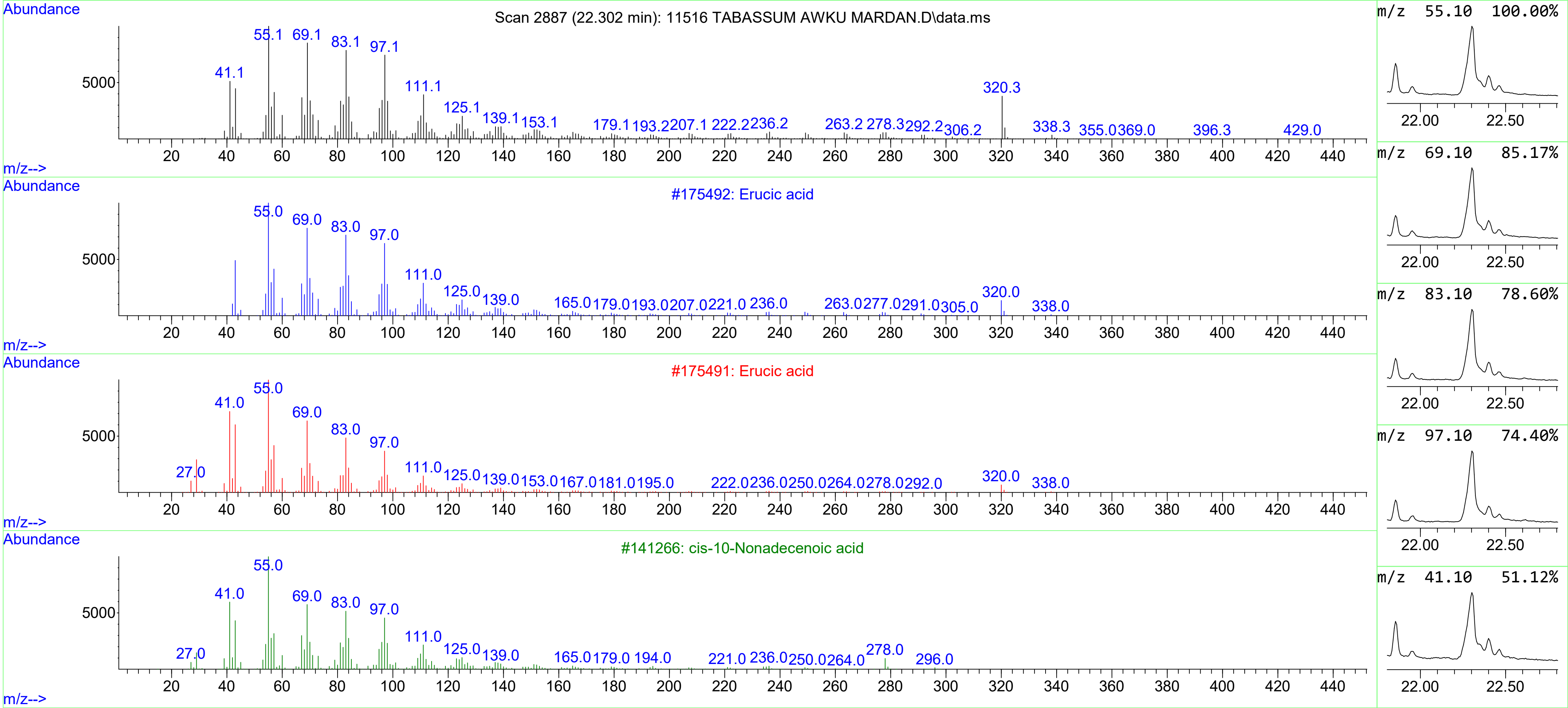

Data File: D:\GCMS RESULTS DATA\11516 TABASSUM AWKU MARDAN.D
Sample : SAMPLE 1

Peak Number: 9 at 22.304 min Area: 227120428 Area % 2.70

The 3 best hits from each library.	Ref\#	CAS\#	Qual
C:\Database\NIST11.L			
1 Erucic acid	175492	000112-86-7	99
2 Erucic acid	175491	000112-86-7	99
3 cis-10-Nonadecenoic acid	141266	073033-09-7	99

Unknown Spectrum based on Apex

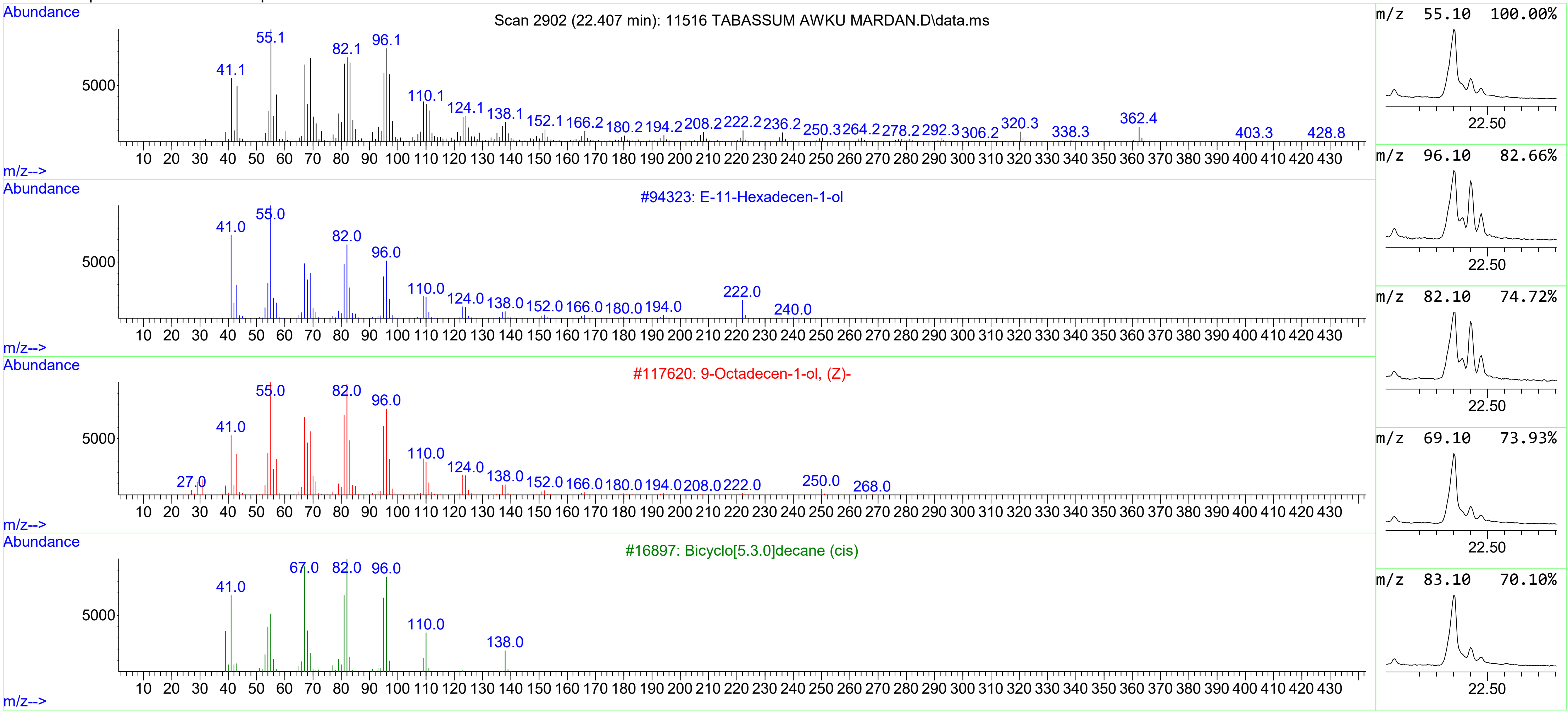

Data File: D:\GCMS RESULTS DATA\11516 TABASSUM AWKU MARDAN.D
Sample : SAMPLE 1

Peak Number: 10 at 22.404 min Area: 76083214 Area % 0.91

The 3 best hits from each library.	Ref\#	CAS\#	Qual
C:\Database\NIST11.L			
1 E-11-Hexadecen-1-ol	94323	1000130-89-8	95
2 9-Octadecen-1-ol, (Z)-	117620	000143-28-2	91
3 Bicyclo[5.3.0]decane (cis)	16897	016189-46-1	86

Unknown Spectrum based on Apex

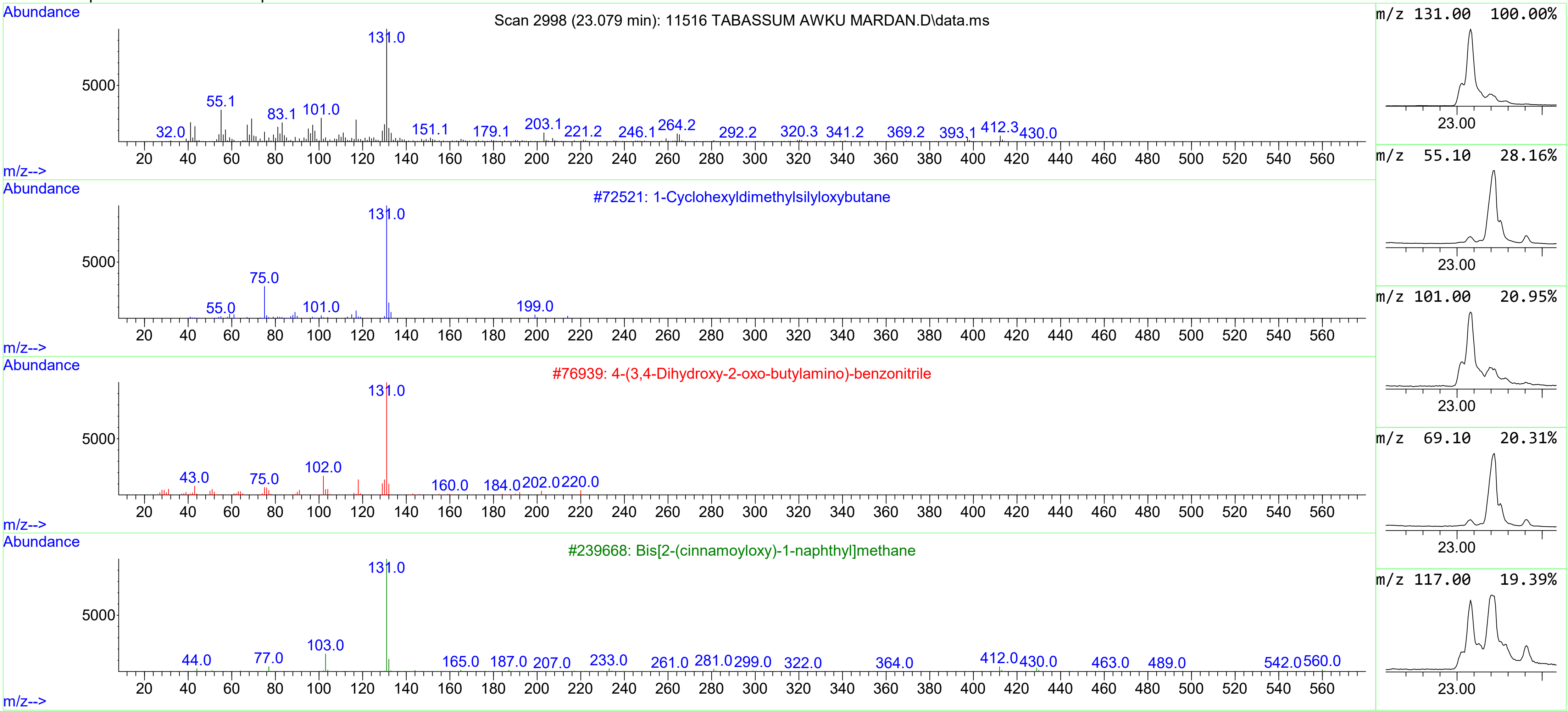

Data File: D:\GCMS RESULTS DATA\11516 TABASSUM AWKU MARDAN.D
Sample : SAMPLE 1

Peak Number: 11 at 23.081 min Area: 77106045 Area % 0.92

The 3 best hits from each library.	Ref\#	CAS\#	Qual
C:\Database\NIST11.L			
1 1-Cyclohexyldimethylsilyloxybutane	72521	1000281-95-6	38
2 4-(3,4-Dihydroxy-2-oxo-butylamin...	76939	1000188-22-9	38
3 Bis[2-(cinnamoyloxy)-1-naphthyl]...	239668	293761-81-6	38

Library Search Report - ChemStation Integrator

Unknown Spectrum based on Apex

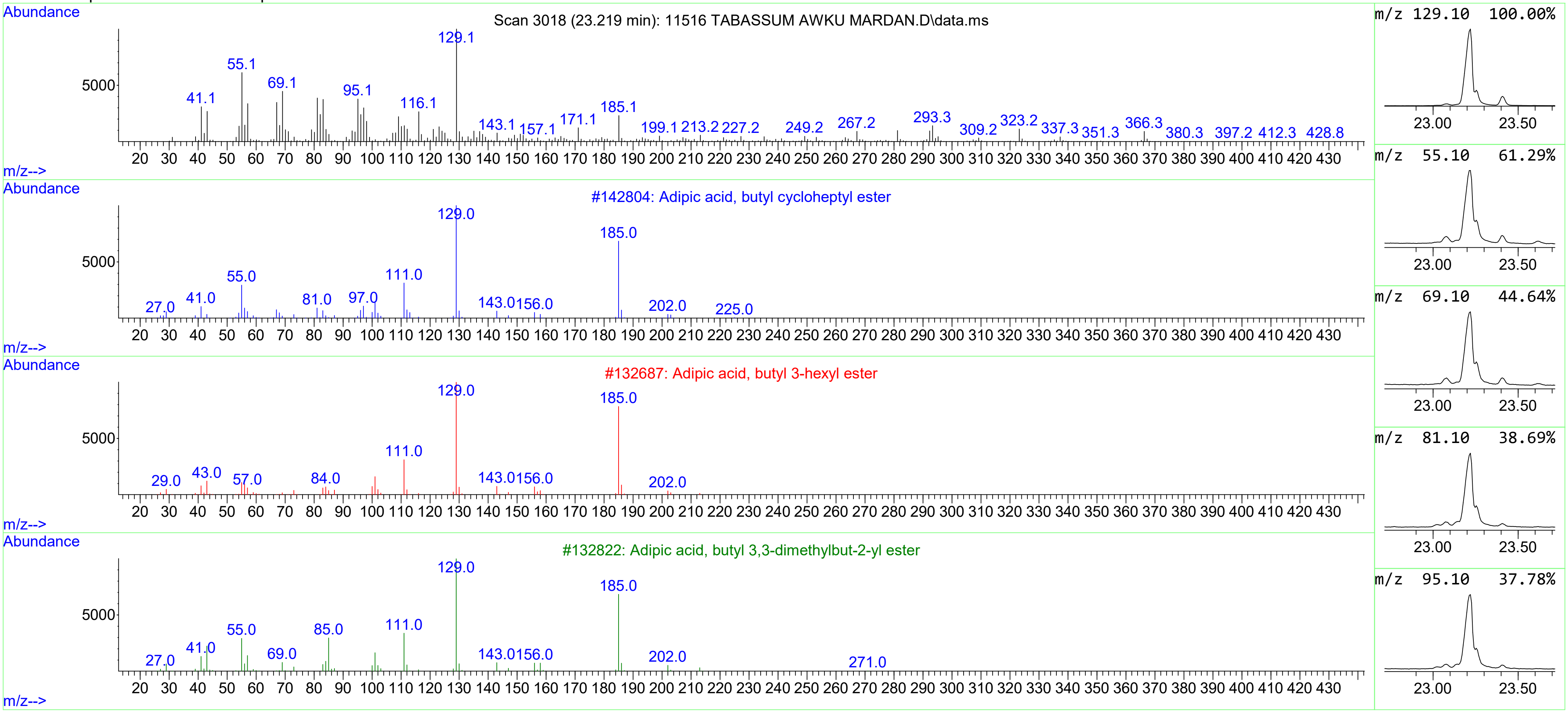

Data File: D:\GCMS RESULTS DATA\11516 TABASSUM AWKU MARDAN.D
Sample : SAMPLE 1

Peak Number: 12 at 23.216 min Area: 638870875 Area % 7.60

The 3 best hits from each library.	Ref#	CAS#	Qual
C:\Database\NIST11.L			
1 Adipic acid, butyl cycloheptyl e...	142804	1000324-71-6	38
2 Adipic acid, butyl 3-hexyl ester	132687	1000353-62-3	35
3 Adipic acid, butyl 3,3-dimethylb...	132822	1000353-66-8	35

Library Search Report - ChemStation Integrator

Unknown Spectrum based on Apex

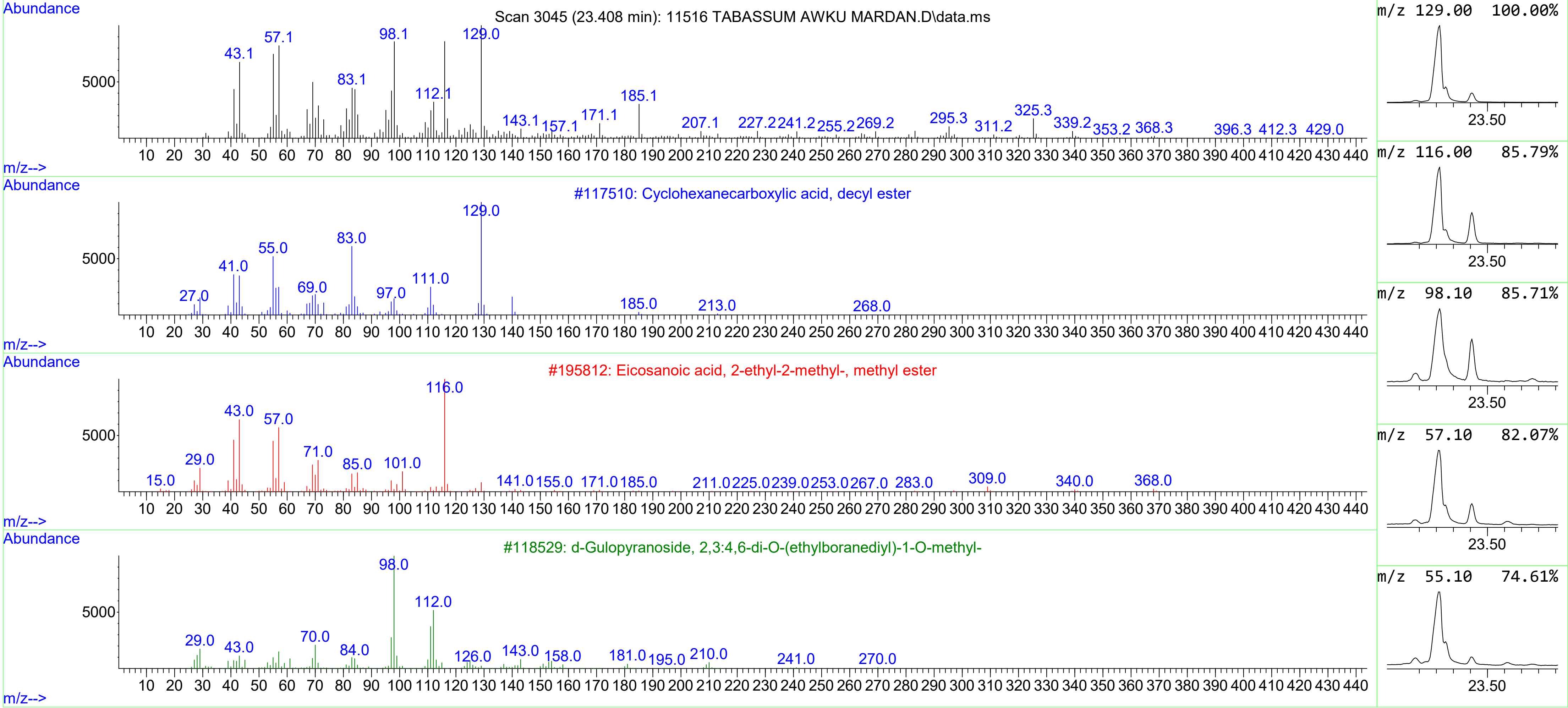

Data File: D:\GCMS RESULTS DATA\11516 TABASSUM AWKU MARDAN.D
Sample : SAMPLE 1

Peak Number: 13 at 23.411 min Area: 57721573 Area % 0.69

The 3 best hits from each library.	Ref\#	CAS\#	Qual
C:\Database\NIST11.L			
1 Cyclohexanecarboxylic acid, decy...	117510	093479-48-2	35
2 Eicosanoic acid, 2-ethyl-2-methy...	195812	055282-04-7	25
3 d-Gulopyranoside, 2,3:4,6-di-O-(...	118529	1000149-94-5	25

Library Search Report - ChemStation Integrator

Unknown Spectrum based on Apex

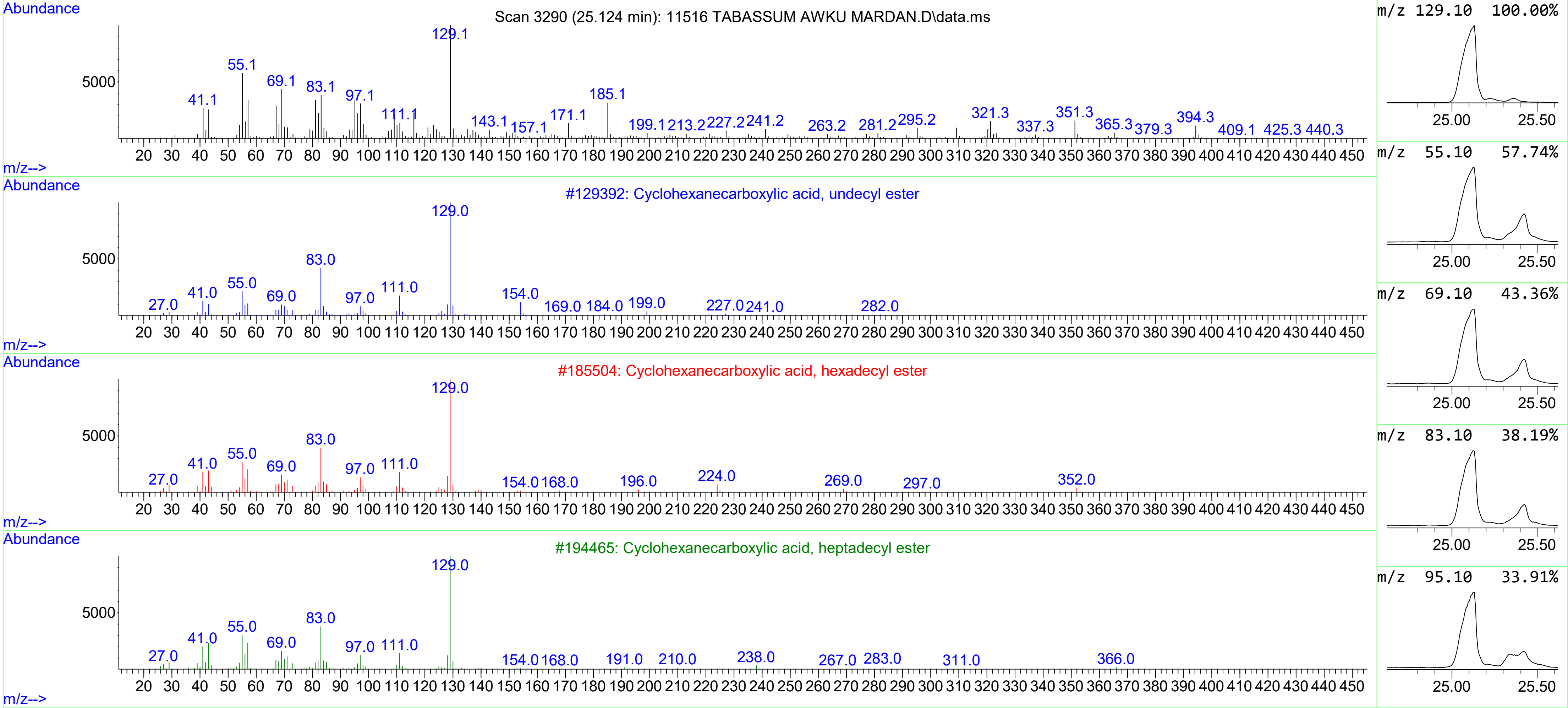

Data File: D:\GCMS RESULTS DATA\11516 TABASSUM AWKU MARDAN.D
Sample : SAMPLE 1

Peak Number: 14 at 25.122 min Area: 1088734677 Area % 12.96

The 3 best hits from each library.	Ref\#	CAS\#	Qual
C:\Database\NIST11.L			
1 Cyclohexanecarboxylic acid, unde...	129392	094107-44-5	42
2 Cyclohexanecarboxylic acid, hexa...	185504	1000279-54-4	42
3 Cyclohexanecarboxylic acid, hept...	194465	1000282-80-3	42

Unknown Spectrum based on Apex

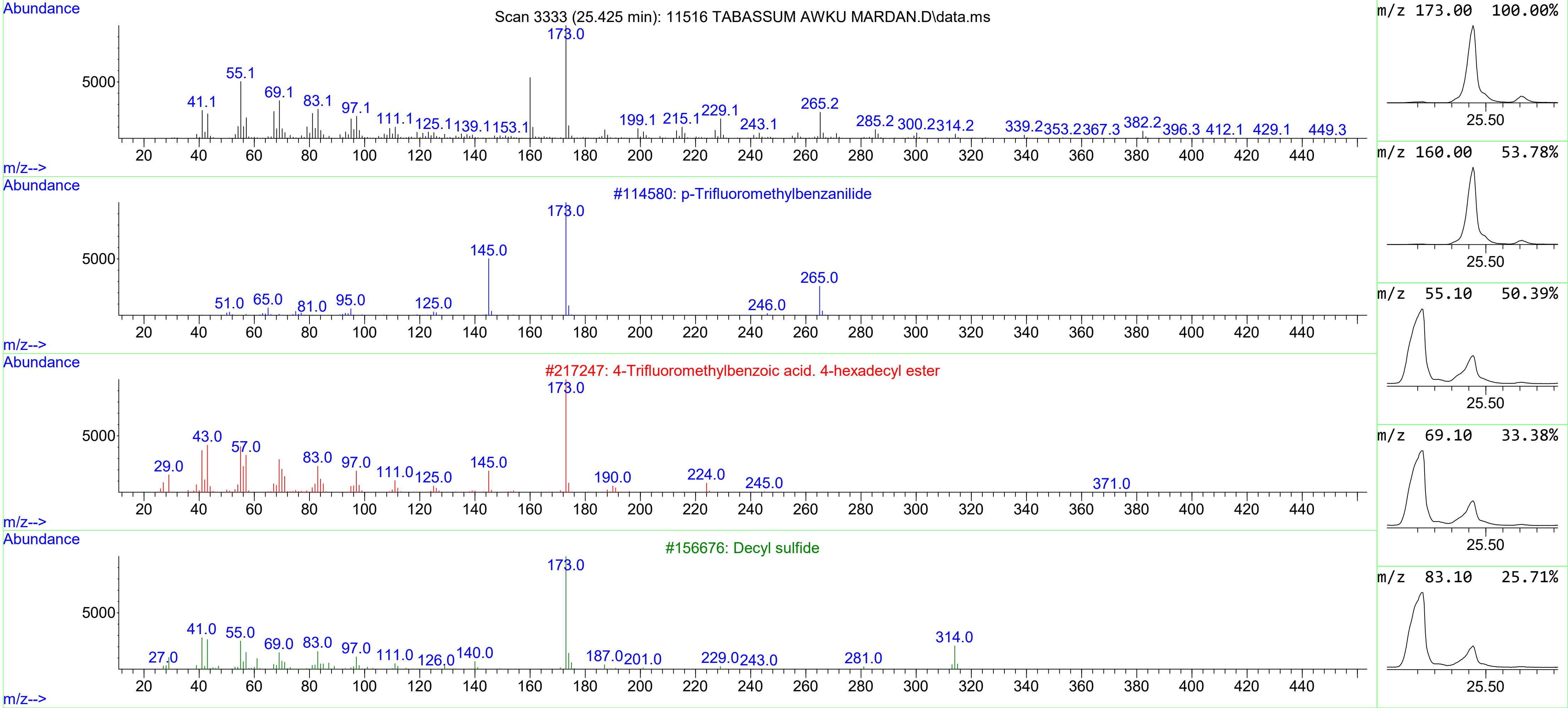

Data File: D:\GCMS RESULTS DATA\11516 TABASSUM AWKU MARDAN.D
Sample : SAMPLE 1

Peak Number: 15 at 25.423 min Area: 378521317 Area % 4.51

The 3 best hits from each library.	Ref\#	CAS\#	Qual
C:\Database\NIST11.L			
1 p-Trifluoromethylbenzanilide	114580	000347-80-8	43
2 4-Trifluoromethylbenzoic acid. 4...	217247	1000283-03-4	38
3 Decyl sulfide	156676	000693-83-4	35

Unknown Spectrum based on Apex

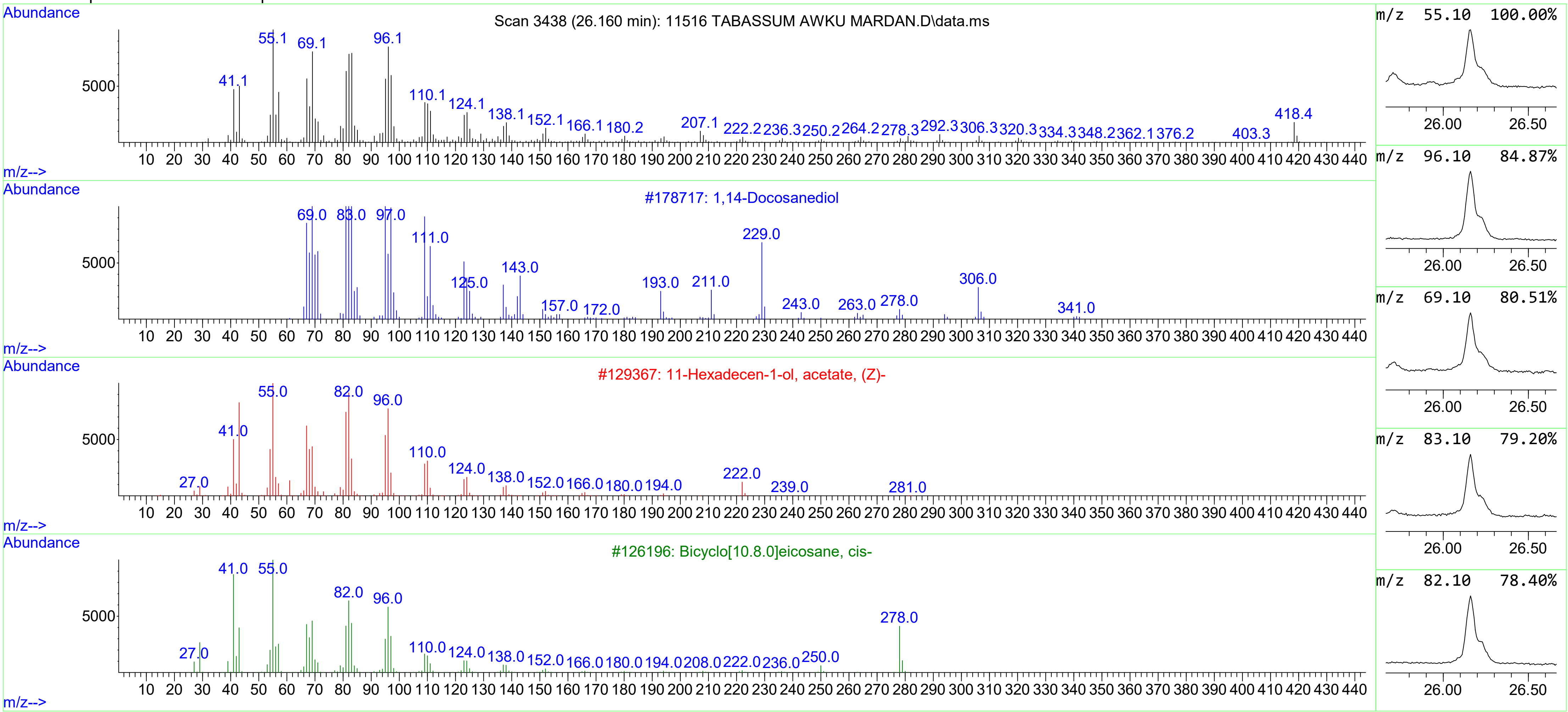

Data File: D:\GCMS RESULTS DATA\11516 TABASSUM AWKU MARDAN.D
Sample : SAMPLE 1

Peak Number: 16 at 26.162 min Area: 65599820 Area % 0.78

The 3 best hits from each library.	Ref\#	CAS\#	Qual
C:\Database\NIST11.L			
1 1,14-Docosanediol	178717	004452-45-3	92
2 11-Hexadecen-1-ol, acetate, (Z)-	129367	034010-21-4	78
3 Bicyclo[10.8.0]eicosane, cis-	126196	1000155-82-2	78

Unknown Spectrum based on Apex

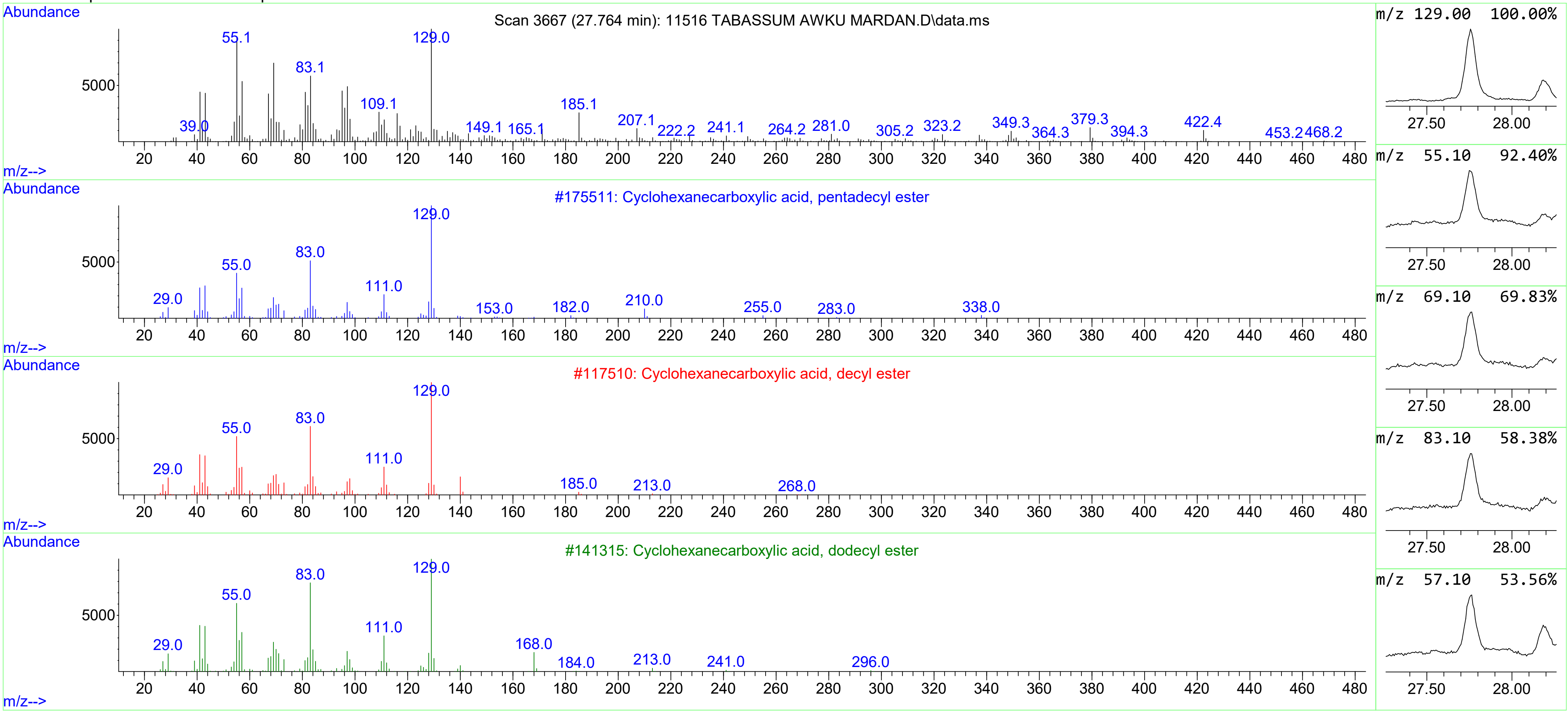

Data File: D:\GCMS RESULTS DATA\11516 TABASSUM AWKU MARDAN.D
Sample : SAMPLE 1

Peak Number: 17 at 27.760 min Area: 51165162 Area % 0.61

The 3 best hits from each library.	Ref\#	CAS\#	Qual
C:\Database\NIST11.L			
1 Cyclohexanecarboxylic acid, pent...	175511	1000279-54-3	49
2 Cyclohexanecarboxylic acid, decy...	117510	093479-48-2	47
3 Cyclohexanecarboxylic acid, dode...	141315	094107-45-6	45

Unknown Spectrum based on Apex

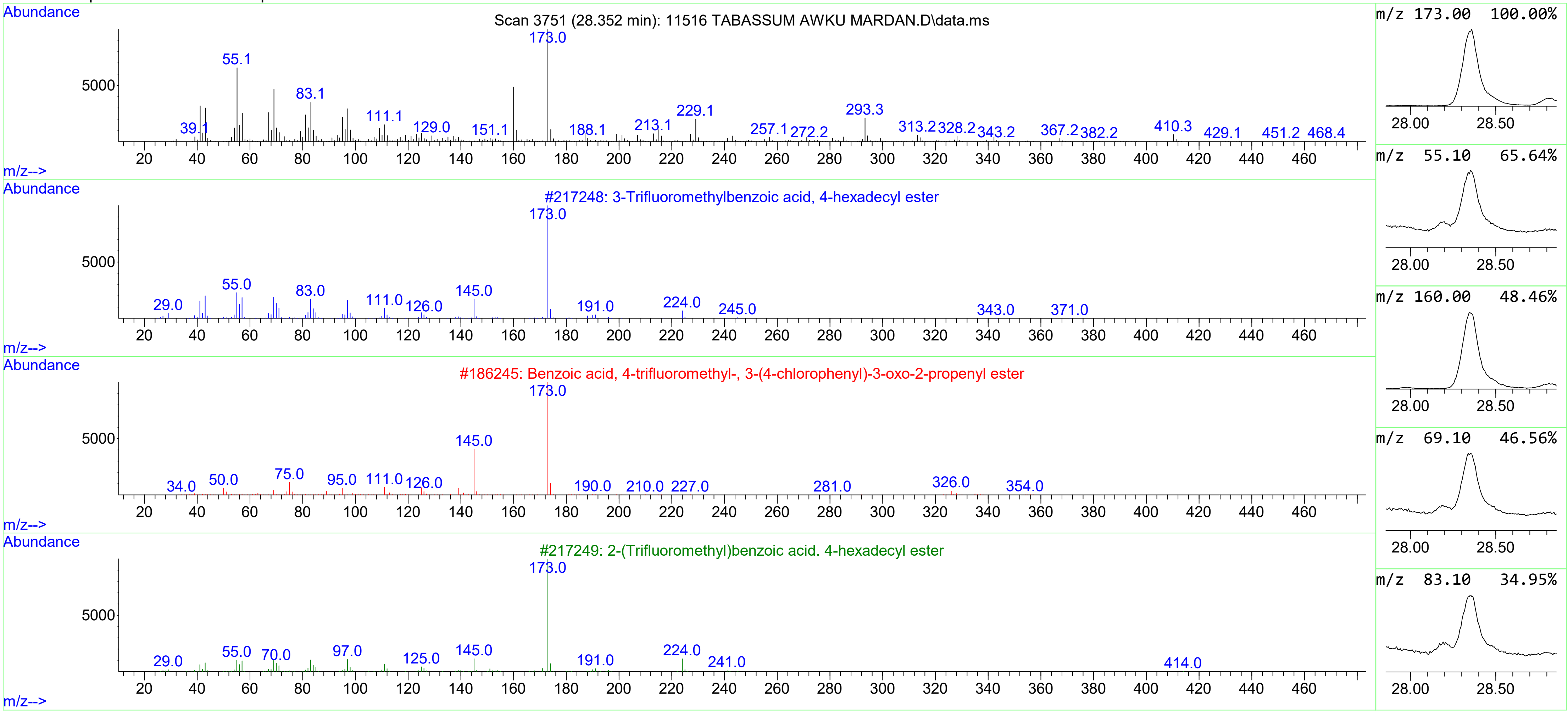

Data File: D:\GCMS RESULTS DATA\11516 TABASSUM AWKU MARDAN.D
Sample : SAMPLE 1

Peak Number: 18 at 28.354 min Area: 107950819 Area % 1.28

The 3 best hits from each library.	Ref\#	CAS\#	Qual
C:\Database\NIST11.L			
1 3-Trifluoromethylbenzoic acid, 4...	217248	1000282-88-0	30
2 Benzoic acid, 4-trifluoromethyl-...	186245	1000267-95-0	30
3 2-(Trifluoromethyl)benzoic acid....	217249	1000283-02-5	27

Unknown Spectrum based on Apex

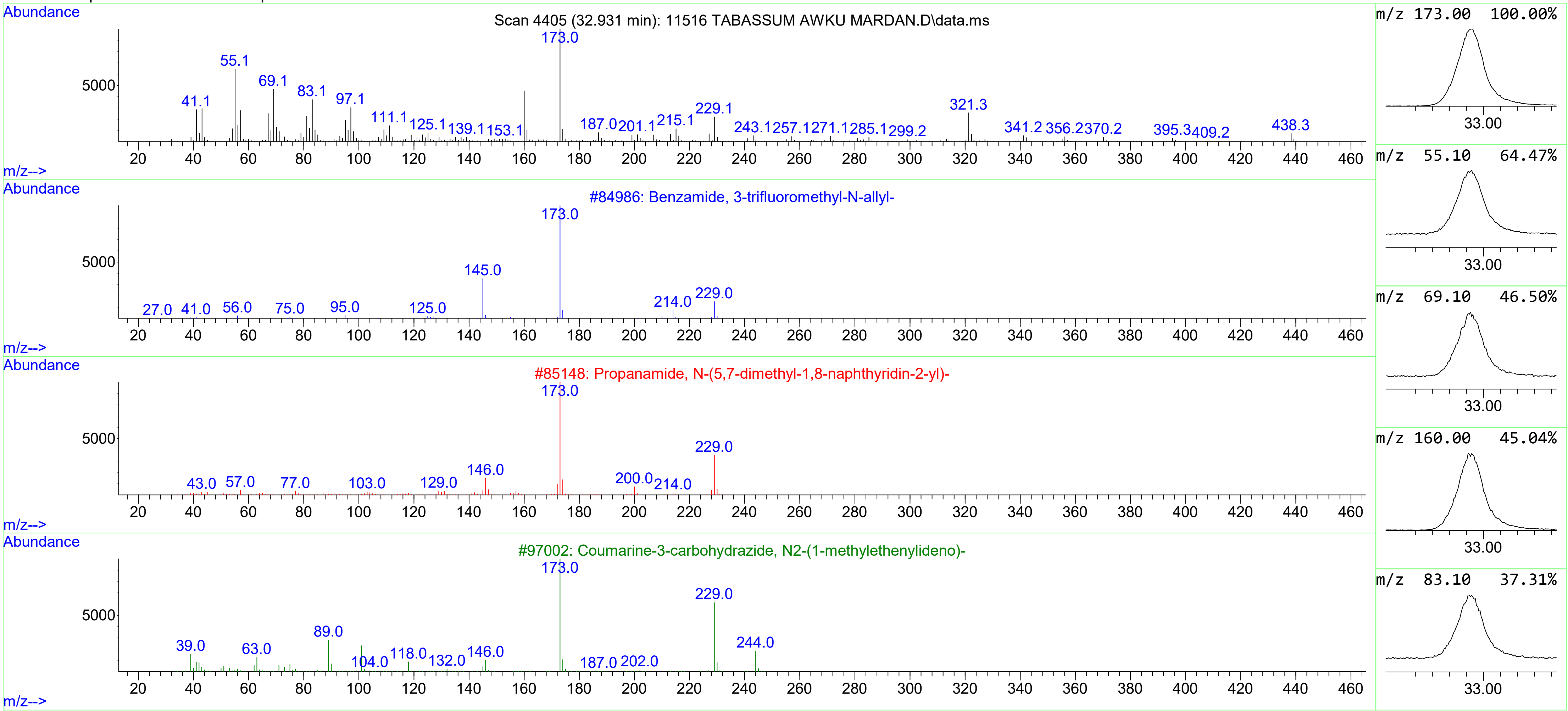

Data File: D:\GCMS RESULTS DATA\11516 TABASSUM AWKU MARDAN.D
Sample : SAMPLE 1

Peak Number: 19 at 32.928 min Area: 170356826 Area % 2.03

The 3 best hits from each library.	Ref\#	CAS\#	Qual
C:\Database\NIST11.L			
1 Benzamide, 3-trifluoromethyl-N-a...	84986	1000340-10-0	35
2 Propanamide, N-(5,7-dimethyl-1,8...	85148	296244-68-3	35
3 Coumarine-3-carbohydrazide, N2-(...	97002	204185-63-7	35
